# The Association of Myosin IB with Actin Waves in Dictyostelium Requires Both the Plasma Membrane-Binding Site and Actin-Binding Region in the Myosin Tail

**DOI:** 10.1371/journal.pone.0094306

**Published:** 2014-04-18

**Authors:** Hanna Brzeska, Kevin Pridham, Godefroy Chery, Margaret A. Titus, Edward D. Korn

**Affiliations:** 1 Laboratory of Cell Biology, National Heart, Lung, and Blood Institute, National Institutes of Health, Bethesda, Maryland, United States of America; 2 Department of Genetics, Cell Biology and Development, University of Minnesota, Minneapolis, Minnesota, United States of America; Université de Genève, Switzerland

## Abstract

F-actin structures and their distribution are important determinants of the dynamic shapes and functions of eukaryotic cells. Actin waves are F-actin formations that move along the ventral cell membrane driven by actin polymerization. *Dictyostelium* myosin IB is associated with actin waves but its role in the wave is unknown. Myosin IB is a monomeric, non-filamentous myosin with a globular head that binds to F-actin and has motor activity, and a non-helical tail comprising a basic region, a glycine-proline-glutamine-rich region and an SH3-domain. The basic region binds to acidic phospholipids in the plasma membrane through a short basic-hydrophobic site and the Gly-Pro-Gln region binds F-actin. In the current work we found that both the basic-hydrophobic site in the basic region and the Gly-Pro-Gln region of the tail are required for the association of myosin IB with actin waves. This is the first evidence that the Gly-Pro-Gln region is required for localization of myosin IB to a specific actin structure *in situ*. The head is not required for myosin IB association with actin waves but binding of the head to F-actin strengthens the association of myosin IB with waves and stabilizes waves. Neither the SH3-domain nor motor activity is required for association of myosin IB with actin waves. We conclude that myosin IB contributes to anchoring actin waves to the plasma membranes by binding of the basic-hydrophobic site to acidic phospholipids in the plasma membrane and binding of the Gly-Pro-Gln region to F-actin in the wave.

## Introduction

Remodeling of the actin cytoskeleton plays an essential role in determining cell shape and movement [1]. Motile eukaryotic cells generate three-dimensional, self-organizing waves of F-actin which propagate along the ventral plasma membrane of cells adhering to a substrate. Identified initially in *Dictyostelium* by Vicker [2], actin waves were subsequently observed in BHK21 fibroblasts and mouse melanoma cells [3], neutrophils [4] and human osteosarcoma cells [5]. Actin waves in *Dictyostelium* have been described in considerable detail, principally by the Gerisch laboratory [6]–[12].

In *Dictyostelium*, actin waves propagate by polymerization of actin filaments at the front and depolymerization of filaments at the rear [7], [8]. When a wave reaches a cell border it may push out a protrusion in the form of a broad lamellipodium and when a wave moves away from the cell border this region often retracts [6], [8]. Because of the similarity in their composition (see below), it has been proposed that actin waves may also function as planar potential phagocytic cup structures that scan a substrate surface in the search of particles to be phagocytosed [10], [11].

Actin waves are more complex in mammalian cells. In neutrophils, actin waves are accompanied by waves of Hem-1 (hematopoietic protein 1) that interacts with the WAVE/SCAR complex that mediates Rac-induced actin polymerization [4]. Reciprocal interactions of the Hem-1 and actin waves appear to be involved in the morphogenesis of motile neutrophils, such as protrusions at the leading edge. In primary mouse fibroblasts, melanoma B16-F10 cells and bone osteosarcoma U2OS cells, actin waves are formed in conjunction with unique integrin-dependent adhesive structures [5]. The “adhesive F-actin waves” contain paxillin, vinculin and talin in addition to integrin, actin and the proteins that regulate actin polymerization [5]. Although it is not clearly established which characteristics of actin waves are shared by *Dictyostelium* and mammalian cells, it is generally agreed that, in both cell types, waves form and move randomly driven by actin polymerization.

Multiple mathematical models describing the formation and propagation of actin waves have been developed, e.g. [13]–[17] and reviewed in [18], but there is little experimental data on the molecular interactions between the several wave components. Understanding the interactions of each component is essential for a full understanding of the structure and function of actin waves. Because of the relatively simple composition of *Dictyostelium* waves compared to mammalian cell waves and the numerous experimental advantages of *Dictyostelium* as a model system for cell motility, in the current study we focused on the interactions between *Dictyostelium* actin waves and myosin IB (MIB), the only myosin that has been shown to be associated with waves.

*Dictyostelium* actin waves contain at least four other cytoskeletal proteins: non-filamentous myosin IB (MIB), Arp2/3, CARMIL and coronin [8], [11]. Myosin II has been shown not to be in waves [9] but the possible presence of other myosins, including other class-I myosins, has not been investigated.

According to a model proposed by Bretschneider et al. [8], the wave consists of a meshwork of branched actin filaments whose barbed ends point to the plasma membrane. MIB occurs throughout the wave but is enriched along the plasma membrane and at the front of the wave. The Arp2/3 complex, which initiates branching of polymerizing actin filaments, occurs throughout the wave but, in contrast to MIB, is more concentrated away from the plasma membrane. CARMIL, a scaffolding protein that binds MIB, Arp2/3 and G-actin, is distributed throughout the wave. Coronin, which inhibits the interaction of Arp2/3 with F-actin and actin polymerization, is enriched at the top of the wave and at the back of the wave where the actin filaments are very short. The actin waves separate two zones on the ventral cell surface [8]–[10]: a zone on one side of the wave that is enriched in Arp2/3, Ras and PIP_3_ and a zone on the other side of the wave that is enriched in myosin II, cortexillin I and PIP_2_ [12].

MIB is a non-filamentous class-I myosin consisting of a single heavy chain and a single light chain [19]. The heavy chain comprises a globular motor-domain (head) that binds F-actin in an ATP-sensitive manner and has actin-activated ATPase activity, followed by a neck (IQ-region) that binds the light chain, and a non-helical tail [20]–[22]. The MIB tail is subdivided into three regions: an N-terminal basic region followed by a Gly-Pro-Gln (GPQ)-rich region and a C-terminal SH3-domain. The basic region of all myosin Is binds acidic phospholipids [20]–[22]. We have recently shown that a short sequence of basic and hydrophobic amino acids (BH-site) within the basic region of *Dictyostelium* MIB is required for MIB to bind to acidic phospholipids *in vitro* [23], [24] and to the plasma membrane *in vivo* [25]. The GPQ-region of MIB binds F-actin in an ATP-insensitive manner [26] which, together with the actin-binding site in the head, allows MIB to crosslink actin filaments [27]. The SH3-domain of MIB binds CARMIL, a scaffolding protein that also binds to Arp2/3, actin capping protein and G-actin [28].

Recently, we reported [25] that the BH site is necessary for binding of MIB to the plasma membrane (where it colocalizes with PIP_3_/PIP_2_) in resting cells, in cell-cell contacts of randomly moving cells, and at the front of motile polarized cells. In addition, the actin-binding site in the head contributes to the re-localization of MIB from uniform distribution on the plasma membrane of resting cells to the plasma membrane at the front of migrating cells. Most likely, the release of MIB from the plasma membrane is facilitated by binding of the head domain to cytoplasmic actin which then concentrates at the front of the motile cell. In that study [25], we found no role for either the GPQ-region or the SH3-domain in the localization of MIB.

To determine the molecular basis of the association of MIB with *Dictyostelium* actin waves, we have now co-expressed GFP-labeled wild-type (WT) MIB and a number of GFP-MIB mutants with mRFP-labeled lifeact, which binds to F-actin, in MIB-null AX2 cells (*myoB^−^*). We find that both the plasma membrane-binding BH-site and the actin-binding GPQ-region in the tail are essential for MIB association with actin waves. Neither the SH3-domain in the tail nor the MIB head are required for binding of MIB to actin waves but a mutation that increases the affinity of the head for F-actin increases MIB association with waves and stabilizes waves.

## Materials and Methods

### DNA constructs

MIB and all its mutant plasmids carried G418 resistance and the expressed myosins had GFP fused at the N-terminus. All but one of the expression plasmids were generated using PCR and PCR-based mutagenesis as described earlier [25]. The plasmid encoding the N154A/BH-Ala mutant was created as follows. A small region of the *myoB* gene carrying the BH-Ala mutation was exchanged into the plasmid carrying the full-length N154A *myoB* gene. The new N154A/BH-Ala *myoB* gene was then ligated into pTX-GFP, a low copy number extrachromosomal GFP expression plasmid [29].

The DNA encoding lifeact [30] with mRFPmars [31] at the C-terminus in the pDM926 plasmid [32] was a generous gift of Dr. D. Veltman (Beatson Institute for Cancer Research, Glasgow, United Kingdom) and was subsequently subcloned between the XhoI and Hind III sites of the pDM358 plasmid [32] that carries hygromycin resistance.

### Cell lines, cell culturing and cell treatment

A blasticidin-resistant strain of *myoB^−^*-cells was made by transforming AX2 cells with a linearized disruption plasmid, pDTB35R, that was generated by inserting the Bsr cassette [33] into the pDTb2 plasmid [34] missing the internal 2.2 kb Bcl fragment. Single colonies were screened for loss of MIB expression by western blotting [34].

AX2 cells were grown in HL5 media [35] and DMIB null cells (*myoB^−^*-cells) were grown in HL5 media with a final concentration of 7 µg/ml blasticidin S HCl (Invitrogen). *myoB^−^* -cells co-expressing lifeact and wild type or mutant MIB were grown in HL5 media with 7 µg/ml blasticidin S HCl, 50 µg/ml hygromycin B (Invitrogen) and 12 µg/ml G418 sulfate (Mediatech). *Dictyostelium* amoebae were grown, as described earlier [25], on 10-cm Petri dishes in HL5 media with appropriate antibiotic additions (see above), harvested in 10 ml of media and placed on ice in 15-ml tubes for 20–30 min. Cells were then plated on chambered cover glass (Nalge Nunc International, 155383) and allowed to attach for 30 min at room temperature.

Cells that were not starved were left in full media and observed for 0.5–3 h after attachment. In all other cases cells were washed 3 times with starvation buffer (10 mM phosphate buffer, pH 6.2, 2 mM MgS0_4_, 0.2 mM CaCl_2_) and observed live at various starvation times in the presence or absence of latrunculin (Latrunculin A, Sigma). Starvation time = 0 corresponds to the time of the first wash. Cells observed in the presence of 1 µM latrunculin were starved 30 min before addition of latrunculin and observed 20 min to 2 h after latrunculin addition.

To induce polarization and streaming, plated cells were kept in starvation buffer at 4°C in the dark overnight and moved to 20°C the following morning. These cells usually formed streams within 3 h after moving them to 20°C. The absence of MIB delays development and over-expression of MIB and expression of some of MIB mutants significantly affects cell behavior [25], [36]–[39]. Therefore, for identifying regions of MIB responsible for its association with actin waves we observed cells in the presence of 1 µM latrunculin at times when they showed similar morphology. We compared cells with similar fluorescence levels and always observed at least two mutants in parallel as controls for each other. Results for each mutant were confirmed in at least two independent transfections and observation of at least 100 cells.

### Other reagents and procedures

*Dictyostelium* cells were transfected with a total of 15 µg of plasmid DNA (7.5 µg of each plasmid for double transfectants) by electroporation (2 times, 0.9 kV) as described [40]. Transformation plates were cultured in the presence of appropriate selecting antibiotics. Cells were viewed with a Zeiss 780 confocal microscope with a 63× lens. The optical-slice thickness was 1 µm unless stated otherwise. Profile scanning of the original cell microscopic images was done using Zeiss Zen software. For final illustrations, images were processed in a Zeiss Zen image browser and Photoshop.

## Results

### Establishing experimental conditions for monitoring MIB association with actin waves

**Figure 1:**
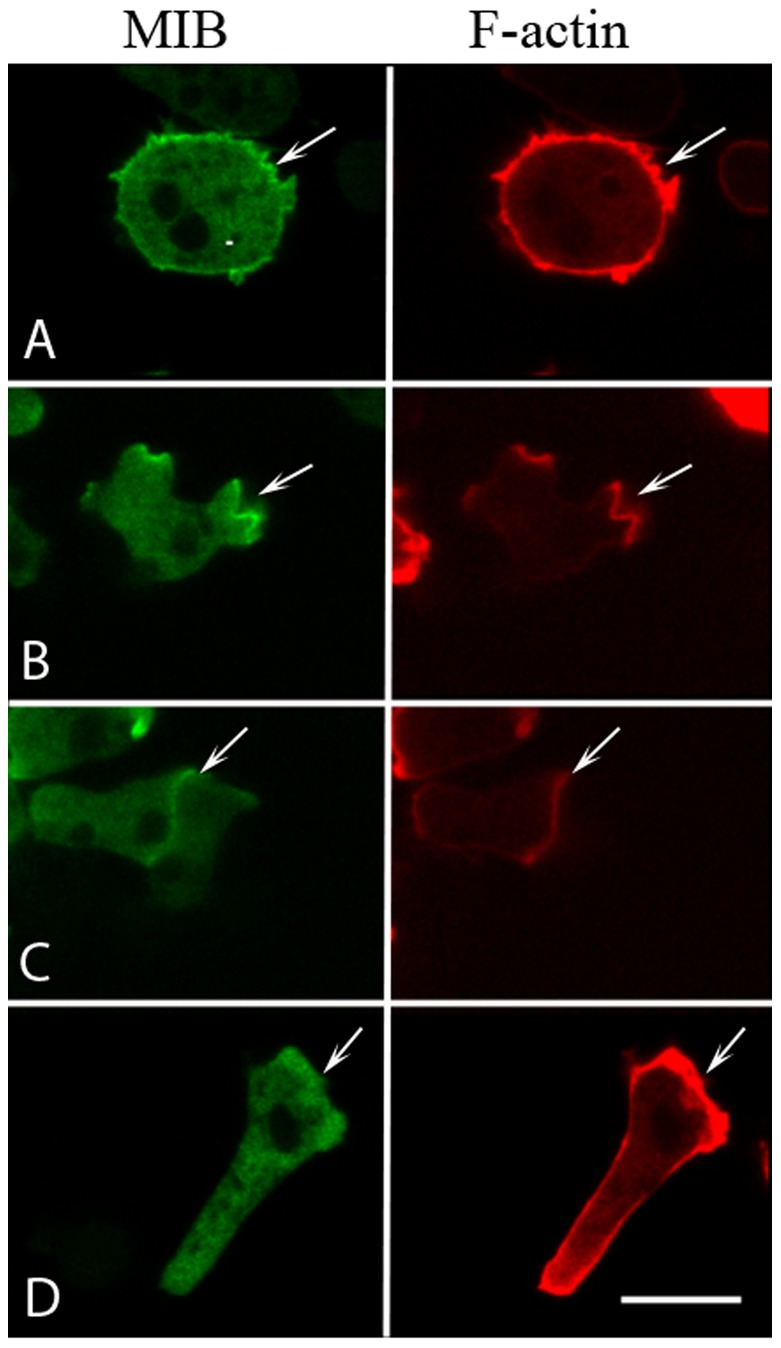
Expression of lifeact does not affect localization of MIB in *myoB^−^*-cells. *myoB^−^*-cells were co-transfected with mRFP-lifeact and GFP-MIB, and the localization of F-actin and MIB were monitored in live cells. MIB co-localized with F-actin: (A) at the plasma membrane of freshly plated cells; (B) in protrusions of cells starved for 1–2 h; (C) at cell-cell contacts of cells starved for 1–2 h; and (D) at the front of elongated cells moving directionally. The arrows point to the sites enriched in MIB. Bar is 10 µm.

We used mRFP-lifeact for monitoring actin waves in all cell lines studied. This allowed us to monitor actin waves independently of MIB associating with them and to compare the association of MIB mutants with waves of similar strength. Before investigating the domain requirements for localization of MIB with actin waves it was important to show that the localization of GFP-MIB co-expressed with mRFP-lifeact in *myoB^−^*-cells was the same as the previously determined localization of endogenous MIB and GFP-MIB expressed in WT-cells. We found that expressed GFP-MIB localized to the plasma membrane of freshly plated cells, to random protrusions and cell-cell contacts in cells starved for a short time, and to the front of elongated cells (Fig. 1), the same as the localization of expressed GFP-MIB in the absence of lifeact and the localization of endogenous MIB [25].

**Figure 2:**
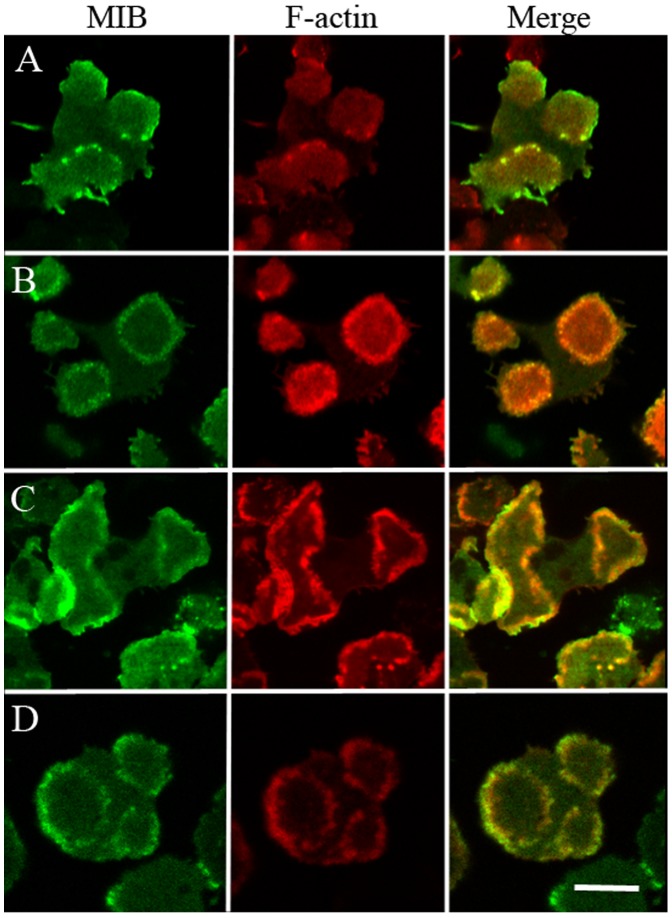
MIB colocalizes with F-actin in waves in cells treated in different ways. GFP-MIB and mRFP-lifeact were expressed in *myoB^−^*-cells in: (A) cells in nutrient media; (B) cells starved for 1 h; (C) cells recovering from treatment with 7.5 µM latrunculin that was washed out, cells were starved before latrunculin treatment; (D) cells in starvation buffer in the presence of 1 µM latrunculin. See Materials and Methods for details of cell treatment. Bar is 10 µm.

It was also necessary to determine the optimal conditions for monitoring the association of MIB with actin waves. As in WT-cells, the *myoB^−^*-cells expressing WT-MIB formed waves under a variety of experimental conditions: cells growing in full media (Fig, 2A) and cells starved for up to 3 h (Fig. 2B); cells treated with ≥5 µM latrunculin [6], which completely depolymerizes actin filaments, after the latrunculin is washed out and actin begins to re-polymerize (Fig. 2C); and cells treated with 1–3 µM latrunculin which induces formation of waves that can be followed for at least 2 h in the presence of latrunculin (Fig. 2D).

**Figure 3:**
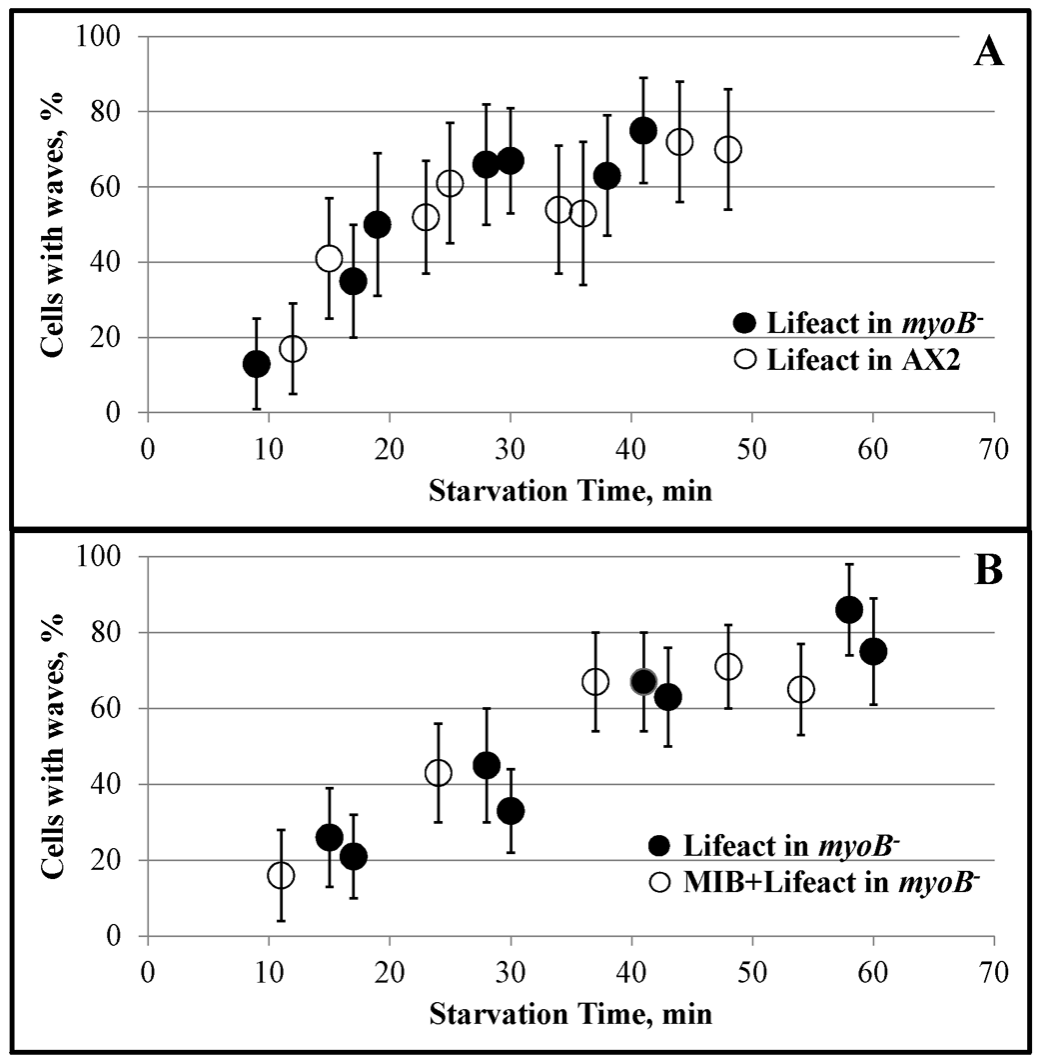
Time-course of the appearance of actin waves in cells starved for short times. The percentages of cells with waves are shown at the starvation times (min) indicated in the figure. Short movies (10 frames every 10 s) of randomly chosen fields were recorded. Cells in each field were scored for the presence of waves visualized with mRFP-lifeact. (A) Comparison of *myoB^−^*-cells (filled circles) and the parent WT-AX2 cells (open circles). Both cell lines were expressing mRFP-lifeact. (B) Comparison of *myoB^−^*-cells expressing either mRFP-lifeact alone (filled circles) or mRFP-lifeact and GFP-MIB (open circles). The two cell lines compared in A and B were grown and treated identically and recorded in the same experiment so the two cell lines within each panel can be directly compared with each other. The results are representative of two independent experiments. Error bars were calculated using the binomial probability confidence interval calculator developed by Daniel S. Soper (http://www.danielsoper.com/statcalc) and correspond to the 95% confidence interval.

The time course of starvation-induced wave formation is quantified in Fig. 3. Only ∼10% of freshly plated WT-AX2 and *myoB^−^*-cells had waves (Fig. 3A) and the percentage of cells with actin waves increased rapidly with starvation reaching 60–80% by 40 min. In this simple assay, there was no difference between the WT and *myoB^−^* -cells (Fig. 3A) or between *myoB^−^* -cells expressing lifeact alone or expressing both lifeact and MIB (Fig. 3B). Either myosin I is not required for wave formation or, perhaps more likely, long-tail myosin IC and/or myosin ID can substitute for MIB (see Discussion).

After prolonged starvation, cells elongate and start moving directionally and eventually form streams. In cells starting to elongate, actin waves were often found at both ends of the cell (Fig. S1, 200 s). The last wave observed in elongating cells was often at the cell front (Fig. S1, 260 s). However, we did not observe actin waves in streaming cells.

Based on the above data, our standard procedure to follow wave formation was to add 1 µM latrunculin to cells that had been starved for 30 min. Under these conditions, a high percentage of cells had waves and the general appearance of the entire cell population remained stable for at least 2 h. Although actin waves have been monitored by both TIRF and confocal microscopy [2]–[4], [6], [8], [9], we used confocal microscopy in our experiments because of its greater flexibility. Also, the high light sensitivity of the Zeiss 780 confocal microscope allowed very low exposure of cells to light, to which *Dictyostelium* is very sensitive; *Dictyostelium* amoebae round up after brief exposure to light, especially after starvation. Since cell fixation partially destroys actin waves and the association of several MIB mutants with waves (not shown), we performed all our studies with live cells.

**Figure 4:**
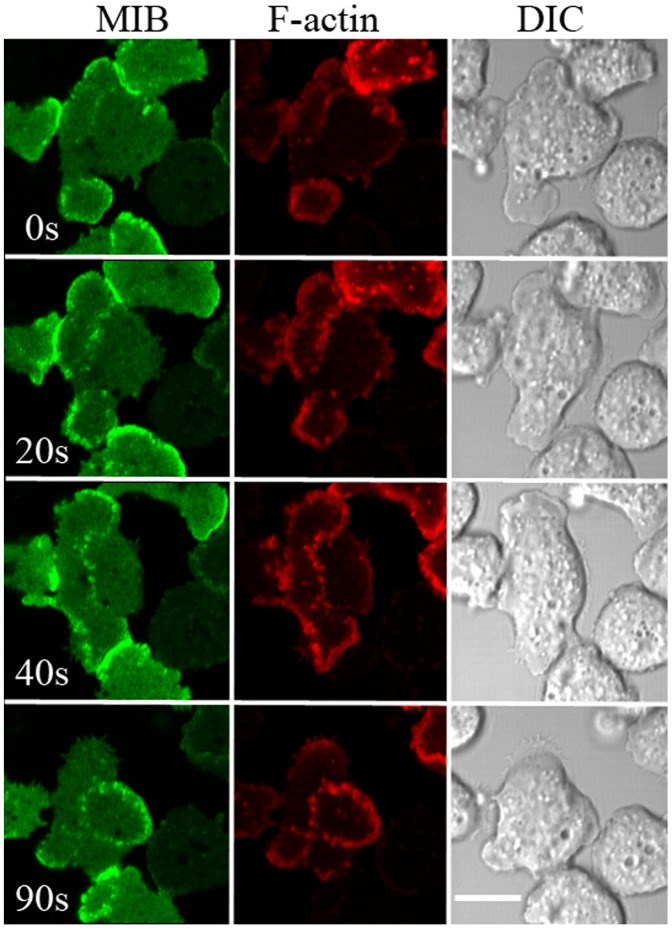
Co-localization of MIB and F-actin in waves; example of different shapes of actin waves formed in the same cells. *myoB^−^*-cells expressing mRFP-lifeact and GFP-MIB were starved for 30 min after which 1 µM latrunculin was added and cell images were recorded at the indicated times (seconds). 0 s corresponds to the beginning of the recording. Bar is 10 µm.

**Figure 5:**
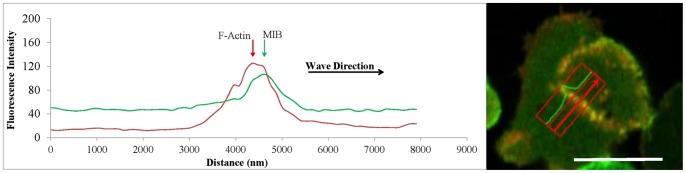
MIB localizes at the front of a moving actin wave in *myoB^−^*-cells expressing GFP-MIB and mRFP-lifeact. The left panel shows the line scans of the fluorescence intensity of GFP-MIB and mRFP-lifeact of the cell shown in the right panel. The line scans correspond to the boxed area within the cell and the red arrow in the box indicates the direction of scanning. 0 nm in the line scan corresponds to the beginning of the box. The direction of wave movement is indicated in the left panel. Cells were starved for 30 min after which 1 µM latrunculin was added and cell images were recorded. Bar is 10 µm.

Importantly, our images of propagating actin waves are similar to those published by others [2], [6], [8], [9]. Fig. 4 shows waves of different shapes formed in the same *myoB^−^*-cells co-transfected with GFP-MIB and mRFP-lifeact. More images of waves of different shapes are shown in Figs. S2 and S3 and in Movies S1 and S2. As reported by Bretschneider et al. [8], MIB was usually more concentrated at the wave front (Fig. 5) and present at the bottom of the wave (see later in Results section). Moreover, the F-actin signal was usually stronger on one side of the wave (Fig. 2), as described by Gerisch et al. [12]. This difference, which corresponds to different modes of F-actin organization [11], was much more pronounced in cells not treated with latrunculin (Fig. 2A).

### MIB mutants

**Figure 6:**
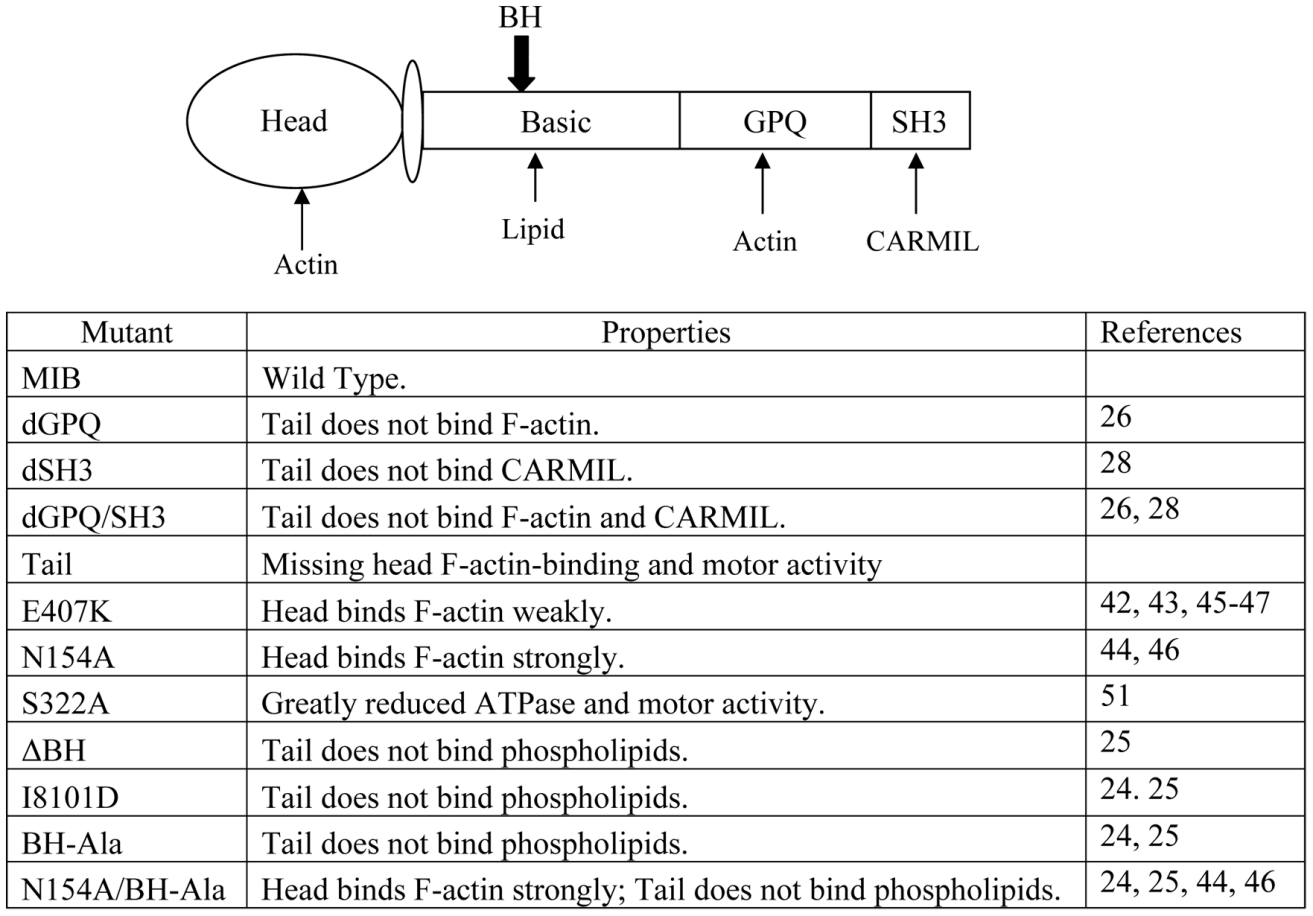
MIB mutants used in the current study.

Having documented that the actin-rich structures that we monitor in the *myoB^−^*-cells and the localization of MIB within them are the same as the actin waves described by others, we could proceed to determine the molecular basis of MIB association with actin waves by expressing a variety of head and tail mutants. The MIB mutants used in this study and their properties are listed in Fig. 6. Most of these mutants and their localizations *in vivo* were described earlier [25]. The GPQ- and SH3-domains in the tail were deleted either separately (dGPQ and dSH3) or together (dGPQ/SH3). The GPQ-domain contains the ATP-insensitive F-actin-binding site [26] which, together with the ATP-sensitive actin-binding site in the head, would allow MIB to crosslink actin filaments [27], as shown previously for *Acanthamoeba* myosin I [41]. The SH3-domain contains the CARMIL-binding site [28]. These three mutants, dGPQ, dSH3 and dGPQ/SH3, bind acidic phospholipids and plasma membrane regions enriched in acidic phospholipids through the BH-site in the basic region of the tail [25]. Mutants in which the BH-site is deleted (dBH) or have a single mutation in the BH-site (I810D) or the five basic residues in the BH-site replaced with Ala (BH-Ala) bind neither acidic phospholipids nor the plasma membrane [24], [25].

Deletion of the head and neck regions (Tail) removes the ATP-sensitive F-actin-binding site and actin-activated ATPase and motor activities. The point mutations in the head-domain are expected to weaken (E407K) or enhance (N154A) binding of MIB to F-actin, by analogy to homologous mutations in *Dictyostelium* myosin II [42]–[44], *Aspergillus* myosin I [45], mammalian MIB [46] and mammalian myosin X [47]. The actin-activated ATPase and motor activities of amoeboid myosin Is require phosphorylation of a Ser or Thr in the head [20], [48]–[50] and mutation of this phosphorylation site in *Dictyostelium* MIB (S322A) substantially reduces both activities and destabilizes the actomyosin complex [51]. The combined BH-Ala and N154A mutant (N154A/BH-Ala) would bind F-actin strongly through its head domain but would not bind to acidic phospholipids or plasma membranes.

### BH-site and GPQ-region in the tail are essential for MIB localization to actin waves

**Figure 7:**
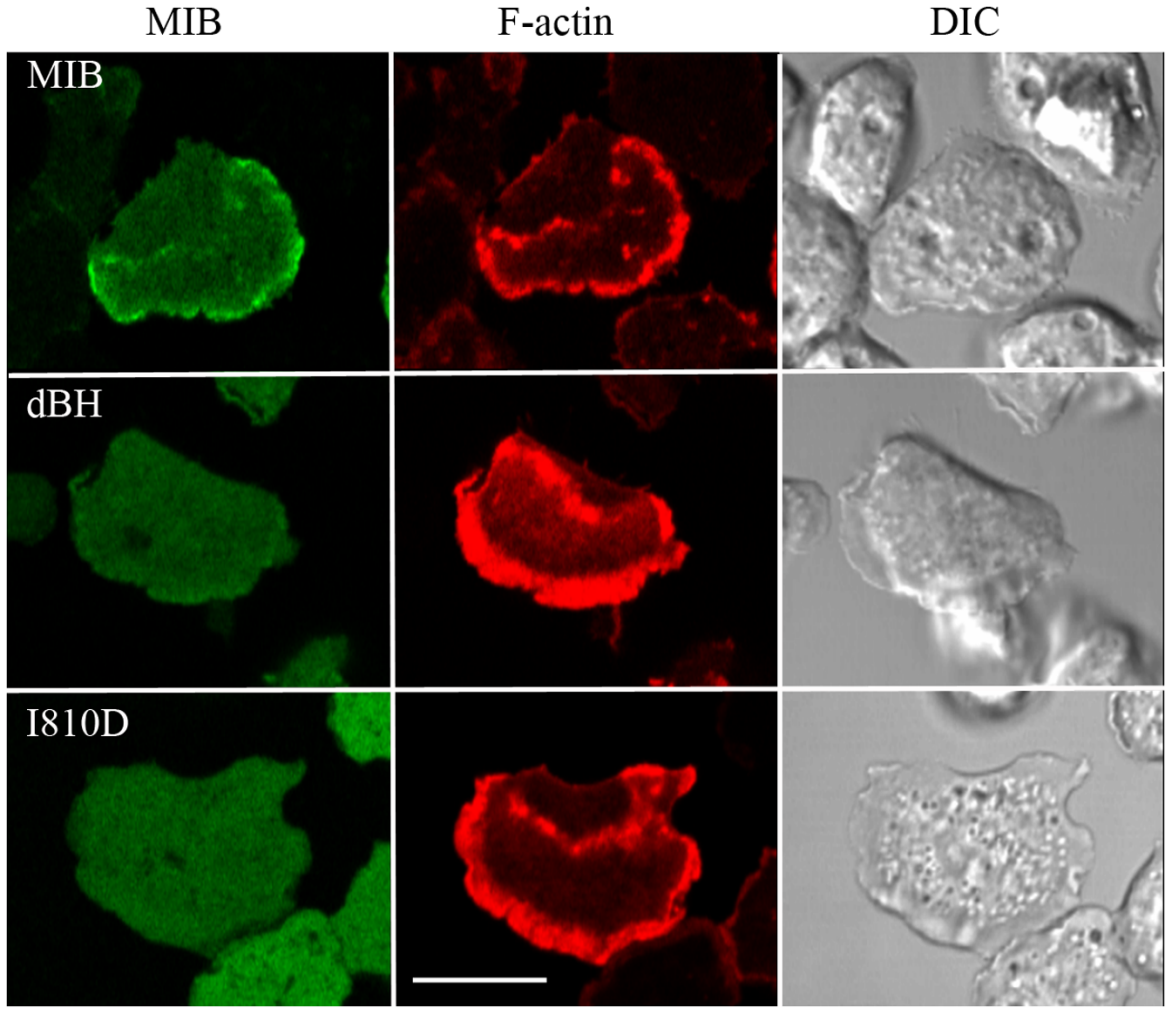
The BH-site is essential for the association of MIB with actin waves. *myoB^−^*-cells were co-transfected with mRFP-lifeact and either GFP-MIB, GFP-dBH or GFP-I810D as indicated in the figure. Neither GFP-dBH nor GFP-I810D binds acidic phospholipids. Cells were starved for 30 min after which 1 µM latrunculin was added and cell images were recorded. MIB associated with the actin wave but dBH and I810D did not. Bar is 10 µm.

**Figure 8:**
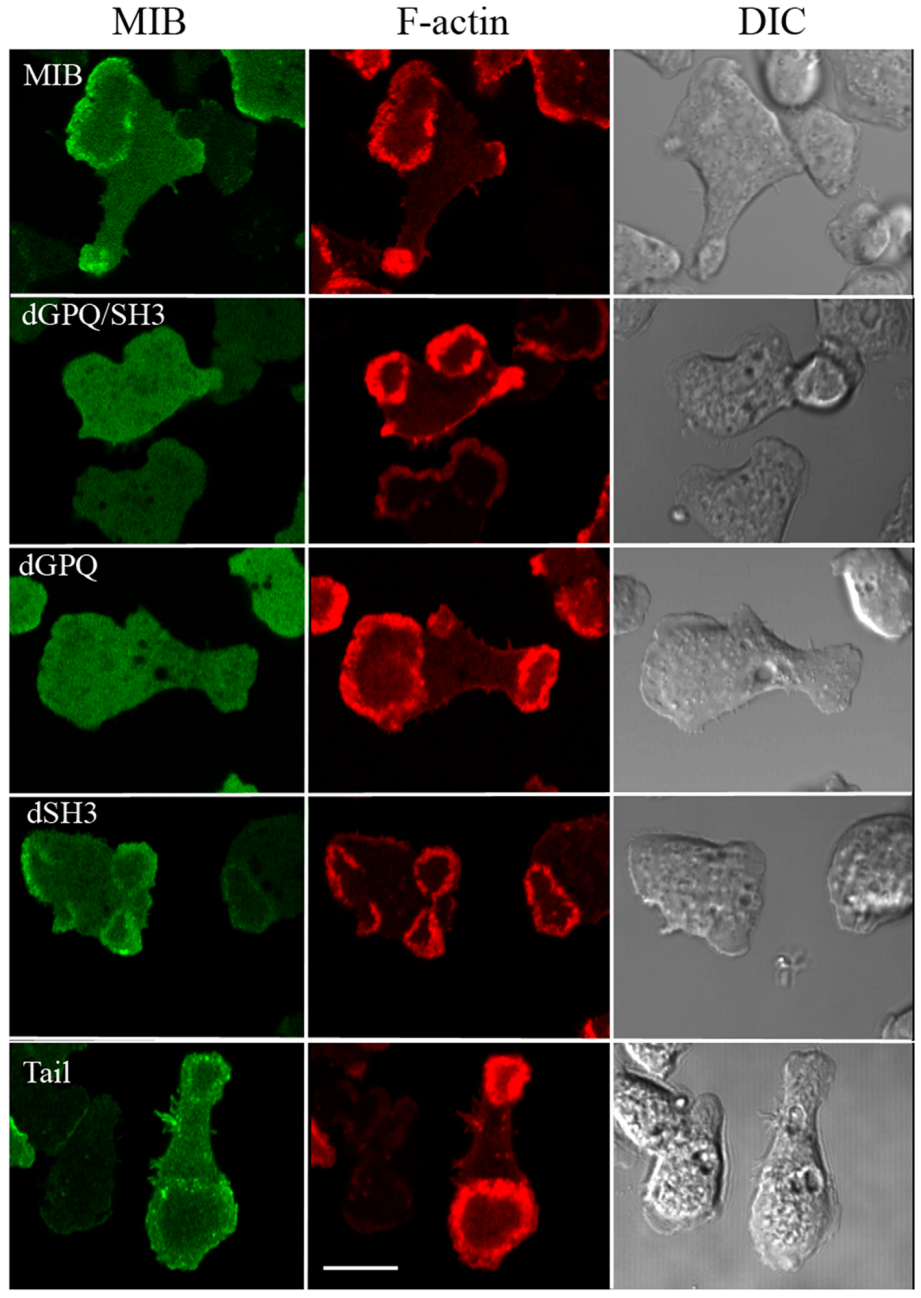
The GPQ-region is essential for MIB association with actin waves. *myoB^−^*-cells were co-transfected with mRFP-lifeact and with either GFP-MIB or GFP-tagged dGPQ/SH3, dGPQ, dSH3 or Tail as indicated in the figure. Cells were starved for 30 min after which 1 µM latrunculin was added and cell images were recorded. Mutants missing the GPQ region (dGPQ and dGPQ/SH3) did not associate with waves whereas dSH3 and Tail did. Bar is 10 µm.

As shown in Fig. 7, two MIB mutants that do not bind acidic phospholipids, dBH and I810D, did not associate with actin waves. This is consistent with the proposal that MIB may serve as an anchor between the plasma membrane and F-actin in the waves [8]. However, the BH-site, although necessary, is not sufficient for binding of MIB to waves. The MIB mutant that contains a wild-type BH-site and binds acidic phospholipids, but does not have the GPQ- and SH3-domains (dGPQ/SH3) also did not associate with actin waves (Fig. 8). In previous experiments [25], this mutant was found to locate to the plasma membrane and cell-cell contacts and relocate to the cell front in chemotaxing cells, but the present results show that binding of MIB to actin waves requires either or both the SH3-domain and/or GPQ-domain.

We found that the mutant missing only the SH3-domain (dSH3) associated with waves whereas the mutant missing only the GPQ-domain (dGPQ) did not associate with waves (Fig. 8). These results demonstrate that the GPQ-domain, which binds to F-actin in the presence or absence of ATP [26], is essential for localization of MIB to the wave and the SH3-domain is not. This the first evidence that the GPQ-domain is required for localization of MIB to a specific actin structure in situ.

Tail alone, which contains the BH-site (plasma membrane-binding) and GPQ-domain (F-actin-binding) but not the head or neck regions of MIB, associated with waves (Fig. 8), but association seemed weaker than for full-length MIB. This might have been because the MIB head contributes to the association of MIB with waves. Therefore, the binding of MIB head mutants to actin waves was assessed.

### Role of head in MIB association with actin waves

**Figure 9:**
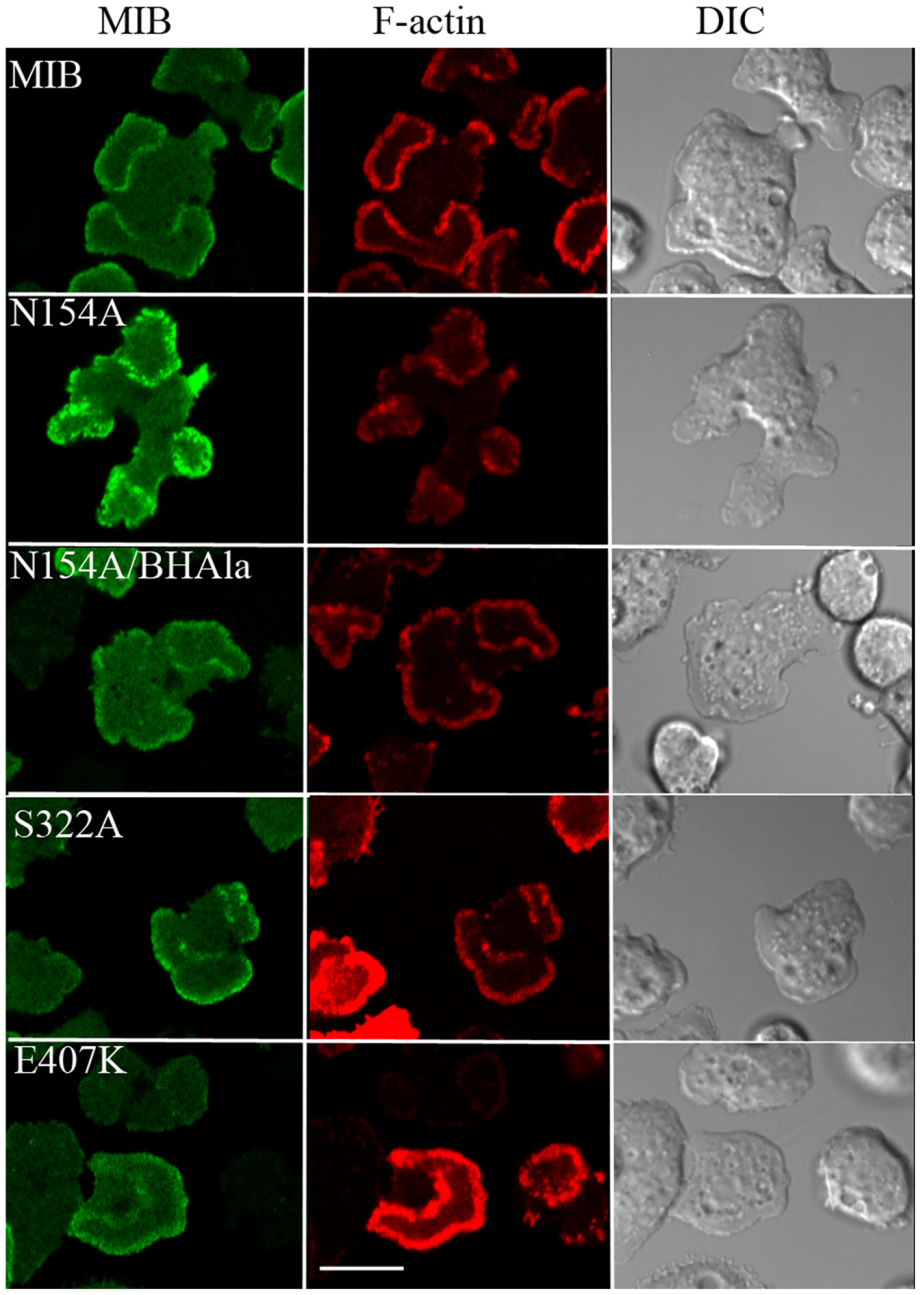
Actin binding through the head contributes to MIB association with waves. *myoB^−^*-cells were co-transfected with mRFP-lifeact and either GFP-MIB or GFP-tagged N154A, N154A/BH-Ala, E407K or S322A, as indicated in the figure. Cells were starved for 30 min after which 1 µM latrunculin was added and cell images were recorded. Bar is 10 µm.

**Figure 10:**
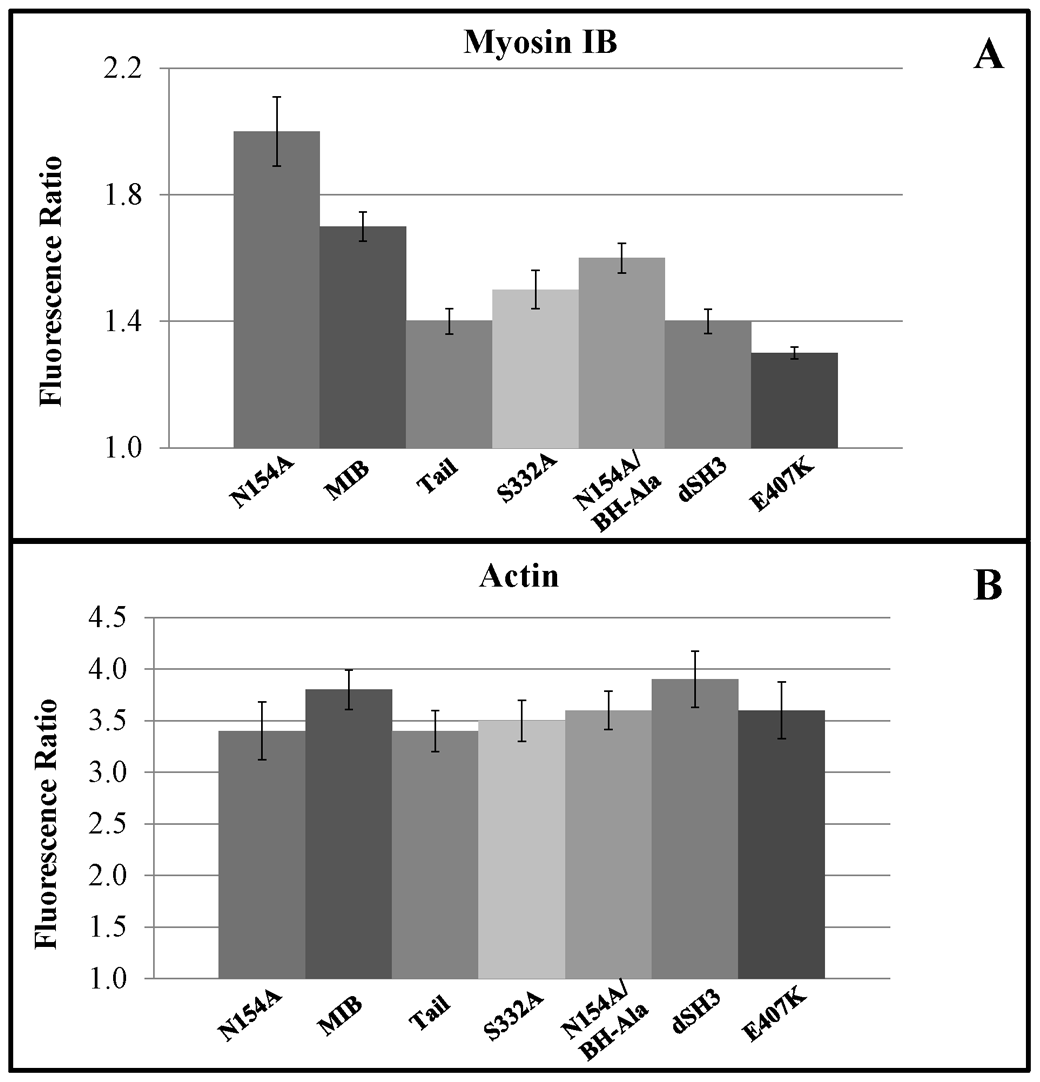
MIB head contributes to wave association; quantification by line scans. *myoB^−^*-cells co-transfected with mRFP-lifeact and GFP-MIB or GFP-tagged MIB mutants were starved for 30 min after which 1 µM latrunculin was added. Cell images were recorded and line scans of images were done as illustrated in Fig. 5. Panel A shows the ratio of maximum fluorescence intensity in the wave to the average fluorescence intensity in the cytoplasm for MIB and its mutants. Panel B shows the same ratio of fluorescence intensity for F-actin in the same waves. Between 16 and 29 cells from 2–4 independent experiments were scanned for each mutant and results were averaged. Standard errors of means are marked in the figure.

The strong actin-binding mutant, N154A, bound to actin waves more strongly than WT-MIB (Fig. 9). Moreover, introducing the N154A mutation into BH-Ala rescued the ability to associate with waves, i.e. N154A/BH-Ala bound to actin waves (Fig. 9) although not as strongly as N154A. Therefore, the enhanced actin binding of N154A targets it to actin waves despite the absence of the membrane binding site. The E407K mutation, which weakens actin-binding by the head, did not abolish association of MIB with waves (Fig. 9) but did weaken association (Fig. 10A). The S322A mutant, which has reduced actin-activated ATPase and motor activities, also associated with actin waves (Fig. 9).

### Quantification of association of MIB mutants with actin waves

**Figure 11:**
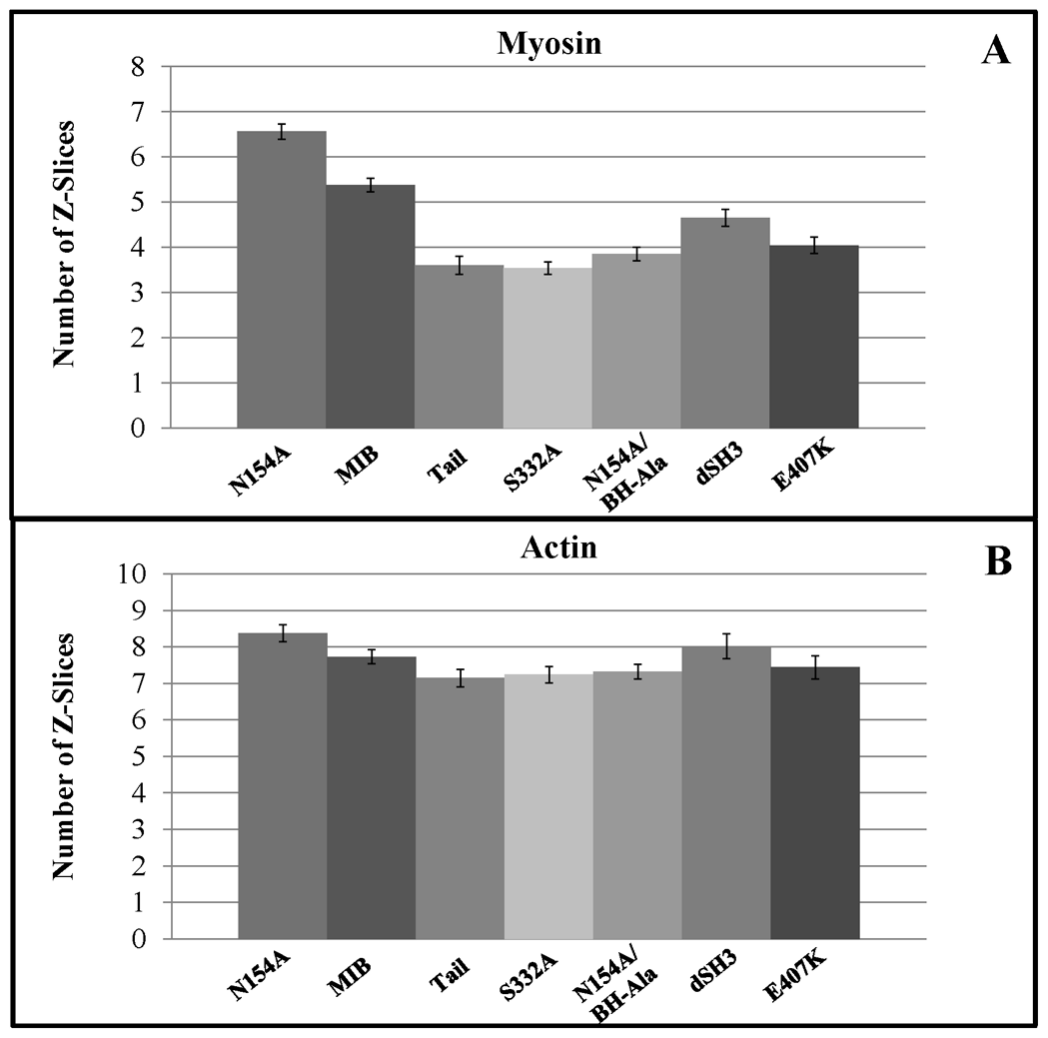
MIB head contributes to wave association; quantification by Z-stacks. *myoB^−^*-cells co-transfected with mRFP-lifeact and GFP-MIB or GFP-tagged MIB mutants were starved for 30 min after which 1 µM latrunculin was added and cell images were recorded at 0.25-µm steps starting at the bottom of the cell. Panel A shows the number of optical slices in which fluorescence of MIB or its mutants were detected in the waves. Panel B shows the number of optical slices in which actin fluorescence was detected in the same waves. Between 25 and 79 images from 2–5 independent experiments were averaged for each mutant. Standard errors of means are shown. Note that actin localization continues to higher focal planes than the localization of MIB and its mutants.

We quantified the association of MIB and its mutants with actin waves by line-scanning images of cells coexpressing mRFP-lifeact and GFP-MIB or its mutants (Fig. 10). We calculated the ratio of the maximum fluorescence intensity of the myosin in the wave to its average intensity in the cytoplasm (Fig. 10A) and calculated the same ratio for lifeact fluorescence in the same wave. For our analysis, we chose cells with similar wave strength, i.e. with similar ratios of lifeact fluorescence (Fig. 10B). Note that the experiments shown in Figs. 10 and 11 were not designed to monitor a potential effect of MIB mutants on the strength of actin waves but only to monitor differences in MIB mutants associations with waves of similar strength. In agreement with our visual assessments, the fluorescence ratio of N154A was substantially higher than the ratio for WT-MIB, and the fluorescence ratios for all of the other MIB mutants were lower than the ratio for WT-MIB (Fig. 10A).

We also recorded Z-stack images of actin waves, starting from the bottom of the cell, and counted the number of optical slices (each 0.25 µm) in which RFP-lifeact and GFP-myosin fluorescence could be detected (Fig. 11). F-actin was detected in 7–8 slices (Fig. 11B), i.e. up to ∼2 µm from the bottom of the cell, whereas MIB was detected only in the lower 5 focal planes (Fig. 11A). These results are consistent with MIB localizing to the lower region of the wave. N154A reached a slightly higher focal plane than WT-MIB, and all the other mutants terminated at slightly lower focal planes than MIB (Fig. 11B).

### Resistance of waves to latrunculin

**Figure 12:**
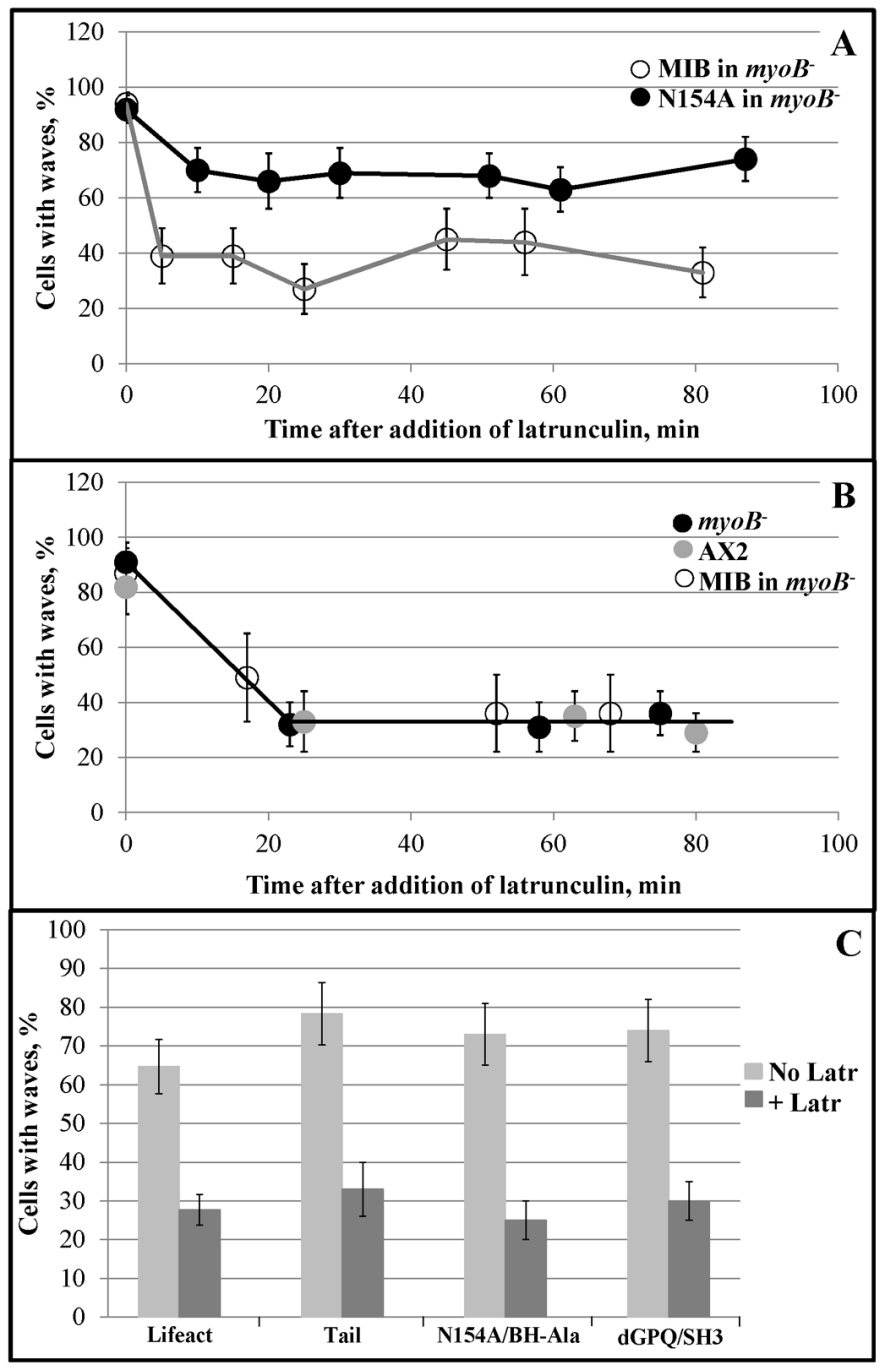
Mutation N154A increases the resistance of actin waves to latrunculin. Cells expressing mRFP-lifeact only or co-expressing mRFP-lifeact and either GFP-MIB or GFP-tagged MIB mutants were starved for 1 h at which time 4.2 µM latrunculin was added. Cells were scored for the presence of actin waves by mRFP-lifeact fluorescence as in Fig. 3. Short movies (10 frames every 10 s) of the fields of cells were taken before and after adding latrunculin and the percent of cells with waves was calculated. (A) *myoB^−^* -cells co-expressing lifeact and MIB (open circles) or lifeact and N154A (black circles). Time = 0 corresponds to cells before addition of latrunculin. (B) *myoB^−^*-cells expressing lifact alone (black circles), parent AX2 cells expressing lifeact alone (grey circles) and *myoB^−^*-cells co-expressing lifeact and MIB (open circles). Time = 0 corresponds to cells before addition of latrunculin. (C) *myoB^−^*-cells expressing lifeact only or co-expressing lifeact and either Tail, N154A-BH-Ala or dGPQSH3 in the absence (grey bars) and presence (black bars) of latrunculin. Movies were recorded before addition of latrunculin (No Latr) and between 60 and 80 min after addition of latrunculin (+Latr) at which time the percentage of cells with waves plateaus. The results are representative of at least 2 independent experiments. Error bars were calculated using the binomial probability confidence interval calculator developed by Daniel S. Soper (http://www.danielsoper.com/statcalc) and correspond to the 95% confidence interval.

The data in Figs. 9–11 show that the association with actin waves is stronger for N154A than for WT-MIB. Although we were unable to show a consistent difference in the percentage of cell forming waves in cells expressing N154A or WT-MIB, we did find that waves in cells expressing N154A were more resistant to latrunculin than waves in cells expressing WT-MIB (Fig. 12A). We observed no difference in resistance of waves to latrunculin between *myoB^−^*-cells, *myoB^−^*-cells expressing MIB and the parent AX2-cells (Fig. 12B), and no difference in the resistance of waves to latrunculin between *myoB^−^*-cells expressing Tail, N154A/BH-Ala, dGPQ/SH3 or lifeact alone (Fig. 12C).

### Regions required for localization of MIB in ventral waves are important for MIB localization to protrusions on the dorsal membrane

Actin waves have been described as planar phagocytic cups [10], [11] because the arrangement of F-actin, PIP_3_, Arp2/3, coronin and MIB are similar in both. We monitored the presence of MIB in actin-rich protrusions formed on the dorsal membrane of *Dictyostelium* cells after 1–3 h of starvation. Since these protrusions did not come in contact with the substrate they are not related to cell movement on the substrate but rather reflect endocytic-related activity of the cells. Although the images were taken at a higher focal plane than used for monitoring waves, by focusing on a lower focal plane we confirmed that, as expected, the monitored cells had waves (Fig. S4).

**Figure 13:**
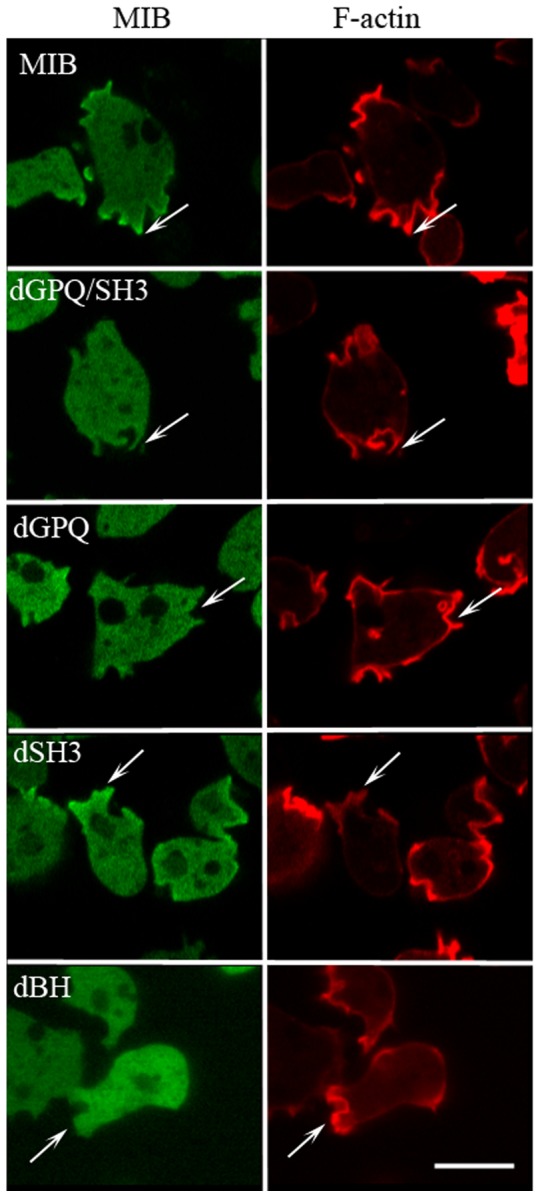
The GPQ-region is required for localization of MIB to protrusions on the dorsal cell membrane. *myoB^−^*-cells co-transfected with mRFP-lifeact and either GFP-MIB or GFP-tagged MIB mutants, as indicated in the figure, were starved for 2 h and monitored for the presence of MIB and its mutants in the actin-rich protrusions on the dorsal cell membrane. These images were taken at a higher focal plane than images visualizing actin waves on the ventral surface. Arrows point to actin-rich protrusions. Bar is 10 µm.

Interestingly, the requirements for localization of MIB mutants to endocytic protrusions were similar to the requirements for their association with actin waves. The fluorescence of WT-MIB was sharp on the border of protrusions (identified by lifeact fluorescence) and was accompanied by more diffuse fluorescence below the edge (Fig. 13). As expected, a MIB mutant incapable of binding acidic phospholipids, dBH, did not localize to protrusions. Also, the mutants that contained the BH-site but not the GPQ-region (dGPQ/SH3 and dGPQ) did not localize to protrusions whereas the mutant missing the SH3-domain (dSH3) did localize sharply to protrusions. However, the differences in localization of MIB mutants to protrusions were a little less striking than for their localization to actin waves; we occasionally observed some diffuse fluorescence beneath protrusion edge for dGPQ and the localization of dSH3 to protrusions seemed slightly weaker than for MIB.

## Discussion

We asked the question, what is the molecular basis of the association of myosin IB with the actin waves that self-propagate along the ventral surface of *Dictyostelium*? We found that two of the three known functional domains in the MIB tail are necessary and sufficient: the GPQ-region, which binds actin, and the BH-site, which binds to the plasma membrane. MIB mutants missing either of these two regions did not associate with waves and deletion of the SH3-domain, the third known functional domain in the MIB tail, did not abolish the binding of MIB to waves. The MIB head-domain, although not required for MIB to bind to actin waves, does contribute as expressed Tail and the mutant with low head-affinity for actin (E407K) bound to waves more weakly than WT-MIB, and the mutant with enhanced head affinity for actin (N154A) associated more strongly with waves than WT-MIB. Enhancing actin affinity of the MIB head compensated for the absence of the BH-site since the N154A/BH-Ala mutant associated with waves and it is possible that the head could play a more important role in association of other myosin Is with waves. Of course, our results do not exclude the possibility that the regions not required for binding of MIB to actin waves may be required for MIB functions (which we did not monitor), e.g. the SH3-domain of MIB may recruit CARMIL to the waves. Although association with CARMIL is not required for MIB recruitment to the wave it might have a stabilizing effect.

The requirement of the BH-site for wave association is in agreement with MIB serving as an anchor between the plasma membrane and F-actin as proposed by Bretschneider et al. [8]. The BH-site binds to regions of the plasma membrane enriched in PIP_2_ and PIP_3_ because their negative charge density is higher than the negative charge of regions containing predominantly phosphatidylserine [23]–[25]. Wave formation starts in the proximity of the plasma membrane and MIB is the wave component located closest to the plasma membrane so the BH-site may act as a wave sensor for local changes in the PIP_2_ and PIP_3_ composition of the membrane.

However, the relation of actin waves to the local concentrations of PIP_2_ and PIP_3_ is not simple. Actin waves can propagate in PTEN (which dephosphorylates PIP_3_)-null cells. i.e. PIP_2_ production is not essential for wave propagation [12]. Also, studies of actin waves and PIP_3_ waves in *Dictyostelium* performed under various conditions [12], [13], [52], [53] have shown that the positions of actin waves do not correspond exactly to the positions of maximal PIP_3_ concentration [53]. In some cases, PIP_3_ waves preceded [13] and, in other cases, followed actin waves [52].

We did not see any obvious effect of deletion of MIB from AX2 cells or of expressing MIB in *myoB^−^*-cells on the ability of cells to form actin waves. Therefore, although MIB is associated with the waves, either a class-I myosin is not essential for wave formation or, perhaps more likely, other class-I myosins compensate for the absence of MIB. *Dictyostelium* has 7 class-I myosins, three of which are “long tailed” and contain potential acidic phospholipid-binding and actin-binding regions in the tail [22]. The possibility of isoform redundancy is supported by the earlier findings that cell lines with multiple deletions of myosin I genes have much stronger phenotypic defects than cell lines missing a single myosin I gene [34], [37], [38], [54]. Also, the published images of double myosin I-null cells show differences in actin distribution at the bottom of the cell suggestive that double myosin I deletions reduces actin wave formation [34], [55]. It may also be that myosin I association with waves serves mainly to relocate myosin I within the cells.

The GPQ-region is known to bind to F-actin and, together with the actin-binding site in the head, may crosslink F-actin filaments. Since MIB could crosslink actin filaments only when the filament concentration is sufficiently high, the GPQ-region in the tail and the actin-binding region in the head may serve as a sensor of local high F-actin concentration. The N154A mutant, that has enhanced head actin affinity and, therefore, enhanced actin crosslinking ability, associates more strongly with waves than WT-MIB and stabilizes waves against disassembly by latrunculin. This most likely reflects actin crosslinking by WT-MIB. Also, the N154A mutation rescues wave association of a mutant with a non-functional plasma membrane-binding site (N154A/BH-Ala). However, enhancement of crosslinking alone is not sufficient for the increased ability of the N154A mutant to stabilize waves. Enhanced actin crosslinking must be combined with the ability to bind to plasma membranes since N154A/BH-Ala, which binds actin strongly through its head but does not bind acidic phospholipids, did not stabilize waves. This observation suggests that crosslinking of actin filaments occurs mostly at the bottom of the waves where MIB can also bind to the plasma membrane.

Since expressed Tail alone localizes to the actin wave, crosslinking actin filaments is not essential for targeting of MIB to the waves. Most probably, the GPQ-region binds directly to F-actin in the waves and, together with the plasma membrane-binding BH-site, anchors actin filaments to the plasma membrane independently of the actin-binding site in the head. As proposed by others [8], the GPQ-region could also contribute to clustering of myosin molecules on the membrane. Also, the GPQ-region may bind some yet unidentified protein other than actin. Since the GPQ-region is proline rich, proteins with SH3-domains would be likely binding partners. Of course, the known and putative functions of the GPQ-region are not mutually exclusive.

We had shown previously [25] that the BH-site is required for localization of expressed MIB to the plasma membrane in freshly plated cells, in cell-cell contacts and in the engulfing mouth of streaming cells. However, the head is required for release of MIB from the plasma membrane and its subsequent relocalization to other sites; i.e. expressed Tail remains associated uniformly with the plasma membrane whereas expressed Head can relocate to the front of chemotaxing cells where, in the absence of the BH-site, it remains diffuse [25]. We concluded then that binding of the head to cytoplasmic F-actin is required for MIB to dissociate from the plasma membrane and, therefore, that both the BH-site in the tail and the actin-binding site in the head are required for proper relocation of MIB during cell movement. Now we show that, in addition to the BH site, the GPQ-region is required for MIB to bind to actin waves, i.e. MIB targeting to different locations in the cell is defined by different regions of the MIB molecule. As F-actin is a binding partner for both the head and the GPQ-region, it may be that the GPQ-region and head distinguish between different arrangements of actin filaments or between different localizations of actin filaments in relation to the plasma membrane and/or other cellular structures.

Finally, we found in the current study that the requirements for localization of MIB to ventral actin waves parallel the requirements for localization of MIB to the endocytic protrusions formed on the dorsal cell membrane after prolonged starvation. In addition, the organization of F- actin and PIP_3_ within actin waves and phagocytic cups are closely related [10], suggesting that targeting of MIB involves specific steric arrangements of actin and phospholipids, and possibly other components. Because of their separation in the Z-direction protrusions are most likely not related directly to waves unless, after reaching the cell border, waves move up the plasma membrane.

## Conclusions

The principal conclusion is that binding of MIB to actin waves in *Dictyostelium* requires both the short basic-hydrophobic plasma membrane-binding site (BH-site) and the actin-binding region (GPQ-region) in the tail. The MIB head-domain is not required but does increase the affinity of MIB to the waves. We have also shown that the two regions that are required for association of MIB to actin waves are also involved in the association of MIB with presumptive endocytic protrusions. This is the first demonstration of a role for the GPQ-region in situ, as only the BH-site is required for association of MIB with other regions of the plasma membrane.

## Supporting Information

Figure S1 **Formation of actin waves in an elongating cell.** *myoB^−^*-cells expressing mRFP-lifeact were starved overnight at 4°C and transferred to room temperature to induce elongation and streaming. Images of an elongating cell are shown recorded at the times (seconds) indicated in the figure. The last observed wave formed at the front of elongating cell. 0 s corresponds to the beginning of recording. Bar is 10 µm.
(TIF)

Figure S2 **Co-localization of N154A and F-actin waves in live cells; example of different shapes of waves formed in the same cells.** *myoB^−^*-cells expressing mRFP-lifeact and GFP-N154A (see Fig. 6) were starved for 30 min after which 1 µM latrunculin was added and cell images were recorded at the indicated times (seconds). 0 s corresponds to the beginning of recording. At all stages N154A co-localized with actin waves. This figure corresponds to Movie S1. Bar is 10 µm.
(TIF)

Figure S3 **Co-localization of N154A and F-actin in actin waves in live cells; formation of a circular wave in the middle of a cell.** *myoB^−^*-cells expressing mRFP-lifeact and GFP-N154A (see Fig. 6) were starved for 30 min after which 1 µM latrunculin was added and cell images were recorded at the indicated times (seconds). 0 s corresponds to the beginning of recording. A circular wave formed in the middle of the cell, merged with a peripheral wave and eventually formed a small circular wave at the cell periphery. N154A co-localized with the F-actin wave at all stages. Fluorescence of N154A was weaker at the early stages of wave formation and gained strength with wave expansion. This figure corresponds to Movie S2. Bar is 10 µm.
(TIF)

Figure S4 **Actin waves and cell protrusions are separated in the Z dimension.** *myoB^−^*cells co-transfected with GFP-MIB and mRFP-lifeact were starved for 2 h and images were taken at the bottom and top (separated by 7 µm) of a live cell. Arrows point to the wave at the cell bottom and to a protrusion at the top of the same cell. Bar is 10 µm.
(TIF)

Movie S1 **Co-localization of N154A and F-actin in live cells; examples of different shapes of waves formed in the middle of a cell.** *myoB^−^*-cells expressing mRFP-lifeact and GFP-N154A mutant were starved for 30 min after which 1 µM latrunculin was added and images were recorded for 200 s (1 frame every 5 s). At all times N154A mutant colocalized with actin waves. This video corresponds to Fig. S2.
(MOV)

Movie S2 **Co-localization of N154A and F-actin in live cells; example of a circular wave formed in the middle of a cell.** *myoB^−^*-cells expressing mRFP-lifeact and GFP-N154A mutant were starved for 30 min after which 1 µM latrunculin was added and images were recorded for 125 s (1 frame every 5 s). A circular wave formed in the middle of the cell, merged with a peripheral wave and eventually formed a small circular wave at the cell periphery. N154A co-localized with the F-actin wave at all stages. Fluorescence of N154A was weaker at the early stages of wave formation and gained strength with wave expansion. This video corresponds to Fig. S3.
(MOV)

## References

[1] Pollard TD, Cooper JA (2005) Actin, a central player in cell shape and movement. Science 326: 1208–1212. 10.1126/science.1175862 PMC3677050 19965462

[2] Vicker MG (2002) F-actin assembly in Dictyostelium cell locomotion and shape oscillations propagates as a self-organized reaction-diffusion wave. FEBS Lett 510: 5–9. 11755520 10.1016/s0014-5793(01)03207-0

[3] Vicker MG (2002) Eukaryotic cell locomotion depends on the propagation of self-organized reaction-diffusion waves and oscillations of actin filament assembly. Exp Cell Res 275: 54–66. 11925105 10.1006/excr.2001.5466

[4] Weiner OD, Marganski WA, Wu LF, Altschuler SJ, Kirschner MW (2007) An actin-based wave generator organizes cell motility. PLoS Biol 5: e221. 17696648 10.1371/journal.pbio.0050221 PMC1945041

[5] Case LB, Waterman CM (2011) Adhesive F-actin waves: a novel integrin-mediated adhesion complex coupled to ventral actin polymerization. PLoS One 6: e26631. 22069459 10.1371/journal.pone.0026631 PMC3206032

[6] Gerisch G, Bretschneider T, Muller-Taubenberger A, Simmeth E, Ecke M, et al (2004) Mobile actin clusters and traveling waves in cells recovering from actin depolymerization. Biophys J 87: 3493–3503. 15347592 10.1529/biophysj.104.047589 PMC1304815

[7] Bretschneider T, Diez S, Anderson K, Heuser J, Clarke M, et al (2004) Dynamic actin patterns and Arp2/3 assembly at the substrate-attached surface of motile cells. Curr Biol 14: 1–10. 14711408 10.1016/j.cub.2003.12.005

[8] Bretschneider T, Anderson K, Ecke M, Muller-Taubenberger A, Schroth-Diez B, et al (2009) The three-dimensional dynamics of actin waves, a model of cytoskeletal self-organization. Biophys J 96: 2888–2 900. 19348770 10.1016/j.bpj.2008.12.3942 PMC3325131

[9] Schroth-Diez B, Gerwig S, Ecke M, Hegerl R, Diez S, et al (2009) Propagating waves separate two states of actin organization in living cells. HFSP J 3: 412–427. 20514132 10.2976/1.3239407 PMC2839813

[10] Gerisch G, Ecke M, Schroth-Diez B, Gerwig S, Engel U, et al (2009) Self-organizing actin waves as planar phagocytic cup structures. Cell AdhMigr 3: 373–382. 10.4161/cam.3.4.9708 PMC2802751 19855162

[11] Gerisch G (2010) Self-organizing actin waves that simulate phagocytic cup structures. PMC Biophys 3: 7. 20298542 10.1186/1757-5036-3-7 PMC2851664

[12] Gerisch G, Ecke M, Wischnewski D, Schroth-Diez B (2011) Different modes of state transitions determine pattern in the phosphatidylinositide-actin system. BMC Cell Biol 12: 42. 21982379 10.1186/1471-2121-12-42 PMC3199247

[13] Taniguchi D, Ishihara S, Oonuki T, Honda-Kitahara M, et al (2013) Phase geometries of two- dimensional excitable waves govern self-organized morphodynamics of amoeboid cells. Proc Natl Acad Sci U S A 110: 5016–5021. 23479620 10.1073/pnas.1218025110 PMC3612638

[14] Carlsson AE (2010) Dendritic actin filament nucleation causes traveling waves and patches. Phys Rev Lett 104: 228102. 20867207 10.1103/PhysRevLett.104.228102 PMC2947330

[15] Whitelam S, Bretschneider T, Burroughs NJ (2009) Transformation from spots to waves in a model of actin pattern formation. Phys Rev Lett 102: 198103. 19519000 10.1103/PhysRevLett.102.198103

[16] Doubrovinski K, Kruse K (2011) Cell motility resulting from spontaneous polymerization waves. Phys Rev Lett 107: 258103. 22243118 10.1103/PhysRevLett.107.258103

[17] Khamviwath V, Hu J, Othmer HG (2013) A Continuum Model of Actin Waves in Dictyostelium discoideum. PLoS One 8: e64272. 23741312 10.1371/journal.pone.0064272 PMC3669376

[18] Carlsson AE (2010) Actin dynamics: from nanoscale to microscale. Annu Rev Biophys 39: 91–110. 20462375 10.1146/annurev.biophys.093008.131207 PMC2967719

[19] Crawley SW, de la Roche MA, Lee SF, Li Z, et al (2006) Identification and characterization of an 8-kDa light chain associated with Dictyostelium discoideum MyoB, a class I myosin. J Biol Chem 281: 6307–6315. 16415352 10.1074/jbc.M508670200

[20] de la Roche MA, Cote GP (2001) Regulation of Dictyostelium myosin I and II. Biochim Biophys Acta 1525: 245–261. 11257438 10.1016/s0304-4165(01)00110-6

[21] Pollard TD, Doberstein SK, Zot HG (1991) Myosin-I. Annu Rev Physiol 53: 653–681. 2042976 10.1146/annurev.ph.53.030191.003253

[22] McConnell RE, Tyska MJ (2010) Leveraging the membrane - cytoskeleton interface with myosin-1. Trends Cell Biol 20: 418–426. 20471271 10.1016/j.tcb.2010.04.004 PMC2897960

[23] Brzeska H, Hwang KJ, Korn ED (2008) Acanthamoeba myosin IC colocalizes with phosphatidylinositol 4,5-bisphosphate at the plasma membrane due to the high concentration of negative charge. J Biol Chem 283: 32014–32023. 18772133 10.1074/jbc.M804828200 PMC2581559

[24] Brzeska H, Guag J, Remmert K, Chacko S, Korn ED (2010) An experimentally based computer search identifies unstructured membrane-binding sites in proteins: application to class I myosins, PAKS, and CARMIL. J Biol Chem 285: 5738–5747. 20018884 10.1074/jbc.M109.066910 PMC2820801

[25] Brzeska H, Guag J, Preston GM, Titus MA, Korn ED (2012) Molecular basis of dynamic relocalization of Dictyostelium myosin IB. J Biol Chem 287: 14923–14926. 22367211 10.1074/jbc.M111.318667 PMC3340229

[26] Rosenfeld SS, Rener B (1994) The GPQ-rich segment of Dictyostelium myosin IB contains an actin binding site. Biochemistry 33: 2322–2328. 8117689 10.1021/bi00174a045

[27] Lee SF, Côté GP (1993) Isolation and characterization of three Dictyostelium myosin –I isozymes. J Biol Chem 268: 20923–20929. 8407927

[28] Jung G, Remmert K, Wu X, Volosky JM, Hammer JA 3rd (2001) The Dictyostelium CARMIL protein links capping protein and the Arp2/3 complex to type I myosins through their SH3 domains. J Cell Biol 153: 1479–1497. 11425877 10.1083/jcb.153.7.1479 PMC2150732

[29] Levi S, Polyakov P, Egelhoff TT (2000) Green fluroescent protein and epitope tag fusion vectors for Dictyostelium discoideum. Plasmid 44: 231–238. 11078649 10.1006/plas.2000.1487

[30] Riedl J, Crevenna AH, Kessenbrock K, Yu JH, Neukirchen D, et al (2008) Lifeact: a versatile marker to visualize F-actin. Nat Methods 5: 605–607. 18536722 10.1038/nmeth.1220 PMC2814344

[31] Fischer M, Haase I, Simmeth E, Gerisch G, Müller-Taubenberger A (2004) A brilliant monomeric red fluorescent protein to visualize cytoskeleton dynamics in Dictyostelium. FEBS Lett 577: 227–232. 15527790 10.1016/j.febslet.2004.09.084

[32] Veltman DM, Akar G, Bosgraaf L, Van Haastert PJ (2009) A new set of small, extrachromosomal expression vectors for Dictyostelium discoideum. Plasmid 61: 110–118. 19063918 10.1016/j.plasmid.2008.11.003

[33] Sutoh K (1993) A transformation vector for Dictyostelium discoideum with a new selectable marker Bsr. Plasmid 30: 150–154. 8234487 10.1006/plas.1993.1042

[34] Novak KD, Peterson MD, Reedy MC, Titus MA (1995) Dictyostelium myosin I double mutants exhibit conditional defects in pinocytosis. J Cell Biol 131: 1205–1221. 8522584 10.1083/jcb.131.5.1205 PMC2120646

[35] Sussman M (1987) Cultivation and synchronous morphogenesis of Dictyostelium under controlled experimental conditions. Methods Cell Biol 28: 9–29. 3298997 10.1016/s0091-679x(08)61635-0

[36] Jung G, Hammer JA 3rd (1990) Generation and characterization of Dictyostelium cells deficient in a myosin I heavy chain isoform. J Cell Biol 110: 1955–1964. 2141028 10.1083/jcb.110.6.1955 PMC2116136

[37] Jung G, Wu X, Hammer JA 3rd (1996) Dictyostelium mutants lacking multiple classic myosin I isoforms reveal combinations of shared and distinct functions. J Cell Biol 133: 305–303. 8609164 10.1083/jcb.133.2.305 PMC2120808

[38] Falk DL, Wessels D, Jenkins L, Pham T, Kuhl S, et al (2003) Shared, unique and redundant functions of three members of the class I myosins (MyoA, MyoB and MyoF) in motility and chemotaxis in Dictyostelium. J Cell Sci 116: 3985–3999. 12953059 10.1242/jcs.00696

[39] Novak KD, Titus MA (1997) Myosin I overexpression impairs cell migration. J Cell Biol 136: 633–647. 9024693 10.1083/jcb.136.3.633 PMC2134295

[40] Gaudet P, Pilcher KE, Fey P, Chisholm R (2007) Transformation of Dictyostelium discoideum with plasmid DNA. Nat Protoc 2: 1317–1324. 17545968 10.1038/nprot.2007.179

[41] Pollard TD, Korn ED (1973) Acanthamoeba myosin. II. Interaction with actin and with a new cofactor protein required for actin activation of Mg^2+^ adenosine triphosphatase activity. J Biol Chem 248: 4691–4697. 4268864

[42] Friedman AL, Geeves MA, Manstein DJ, Spudich JA (1998) Kinetic characterization of myosin head fragments with long-lived myosin.ATP states. Biochemistry 37: 9679–9687. 9657680 10.1021/bi973143f

[43] Ruppel KM, Spudich JA (1996) Structure-function studies of the myosin motor domain: importance of the 50-kDa cleft. Mol Biol Cell 7: 1123–1136. 8862525 10.1091/mbc.7.7.1123 PMC275963

[44] Shimada T, Sasaki N, Ohkura R, Sutoh K (1997) Alanine scanning mutagenesis of the switch I region in the ATPase site of Dictyostelium discoideum myosin II. Biochemistry 36: 14037–14043. 9369475 10.1021/bi971837i

[45] Liu X, Osherov N, Yamashita R, Brzeska H, Korn ED, et al (2001) Myosin I mutants with only 1% of wild-type actin-activated MgATPase activity retain essential in vivo function(s). Proc Natl Acad Sci U S A 98: 9122–9127. 11459943 10.1073/pnas.161285698 PMC55383

[46] Almeida CG, Yamada A, Tenza D, Louvard D, Raposo G, et al (2011) Myosin 1b promotes the formation of post-Golgi carriers by regulating actin assembly and membrane remodelling at the trans-Golgi network. Nat Cell Biol 13: 779–789. 21666684 10.1038/ncb2262

[47] Kerber ML, Jacobs DT, Campagnola L, Dunn BD, Yin T, et al (2009) A novel form of motility in filopodia revealed by imaging myosin-X at the single-molecule level. Curr Biol 19: 967–973. 19398338 10.1016/j.cub.2009.03.067 PMC2817954

[48] Brzeska H, Lynch TJ, Martin B, Korn ED (1989) The localization and sequence of the phosphorylation sites of Acanthamoeba myosins I. An improved method for locating the phosphorylated amino acid. J Biol Chem 264: 19340–19348. 2530230

[49] Brzeska H, Korn ED (1996) Regulation of class I and class II myosins by heavy chain phosphorylation. J Biol Chem 271: 16983–16986. 8707782 10.1074/jbc.271.29.16983

[50] Bement WM, Mooseker MS (1995) TEDS rule: a molecular rationale for differential regulation of myosins by phosphorylation of the heavy chain head. Cell Motil Cytoskeleton 31: 87–92. 7553910 10.1002/cm.970310202

[51] Tsiavaliaris G, Fujita-Becker S, Durrwang U, Diensthuber RP, Geeves MA, et al (2008) Mechanism, regulation, and functional properties of Dictyostelium myosin-1B. J Biol Chem 283: 4520–4527. 18089562 10.1074/jbc.M708113200

[52] Asano Y, Nagasaki A, Uyeda TQ (2008) Correlated waves of actin filaments and PIP3 in Dictyostelium cells. Cell Motil Cytoskeleton 65: 923–349. 18814278 10.1002/cm.20314

[53] Gerisch G, Schroth-Diez B, Muller-Taubenberger A, Ecke M (2012) PIP3 waves and PTEN dynamics in the emergence of cell polarity. Biophys J 103: 1170–1178. 22995489 10.1016/j.bpj.2012.08.004 PMC3446687

[54] Ostap EM, Pollard TD (1996) Overlapping functions of myosin-I isoforms? J Cell Biol 133: 221–224. 8609156 10.1083/jcb.133.2.221 PMC2120801

[55] Peterson D, Titus MA (1994) F-actin distribution of Dictyostelium myosin I double mutants. J Eukaryot Microbio 41: 652–657. 10.1111/j.1550-7408.1994.tb01529.x 7866390

