# Supplementary figures and images for: The Association of Myosin IB with Actin Waves in Dictyostelium Requires Both the Plasma Membrane-Binding Site and Actin-Binding Region in the Myosin Tail
Source: PLoS One. 2014 Apr 18;9(4):e94306. doi: 10.1371/journal.pone.0094306 (PMC3991602; doi:10.1371/journal.pone.0094306)

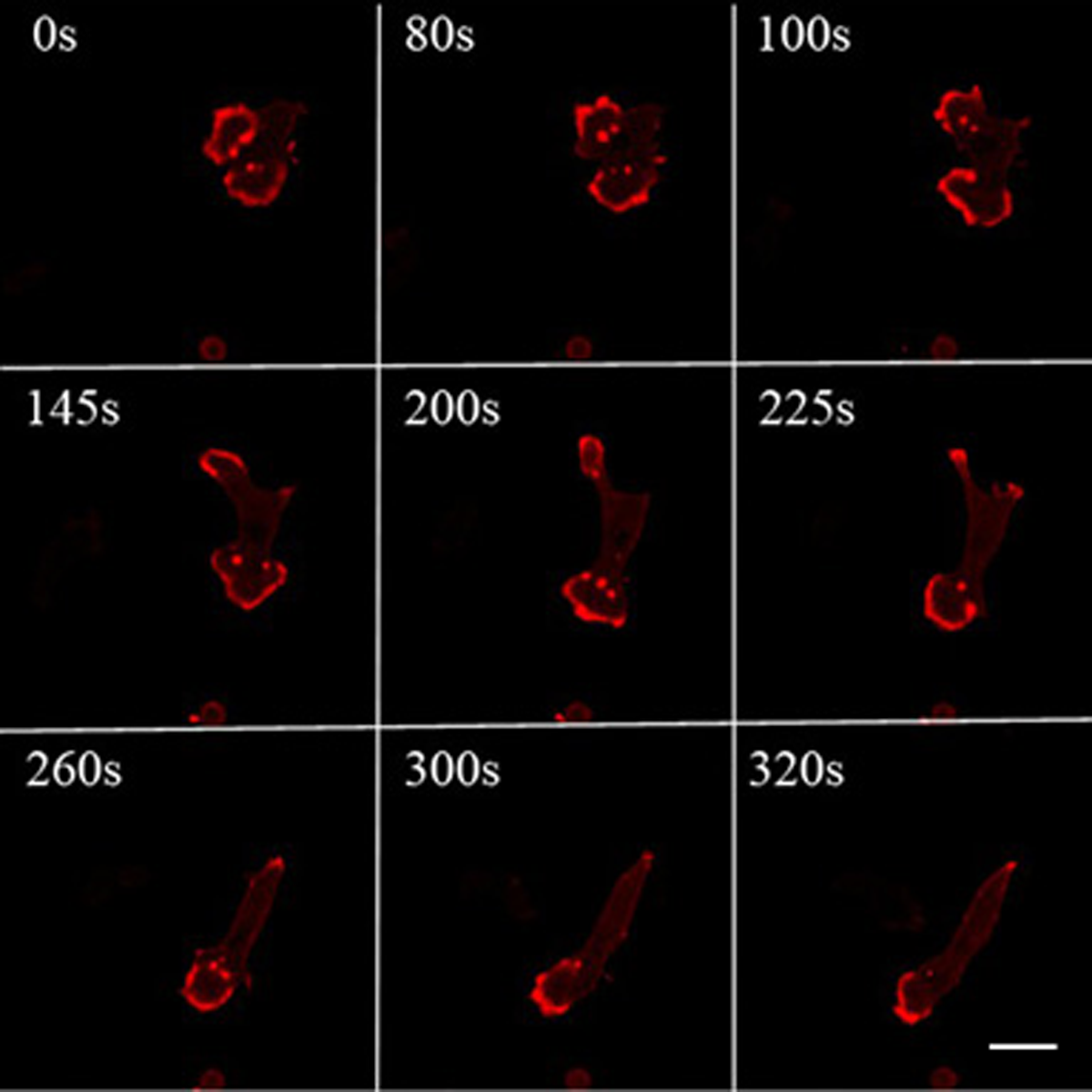

Supplement: Figure S1 — Formation of actin waves in an elongating cell. myoB−-cells expressing mRFP-lifeact were starved overnight at 4°C and transferred to room temperature to induce elongation and streaming. Images of an elongating cell are shown recorded at the times (seconds) indicated in the figure. The last observed wave formed at the front of elongating cell. 0 s corresponds to the beginning of recording. Bar is 10 µm. (TIF) [file pone.0094306.s001.tif]

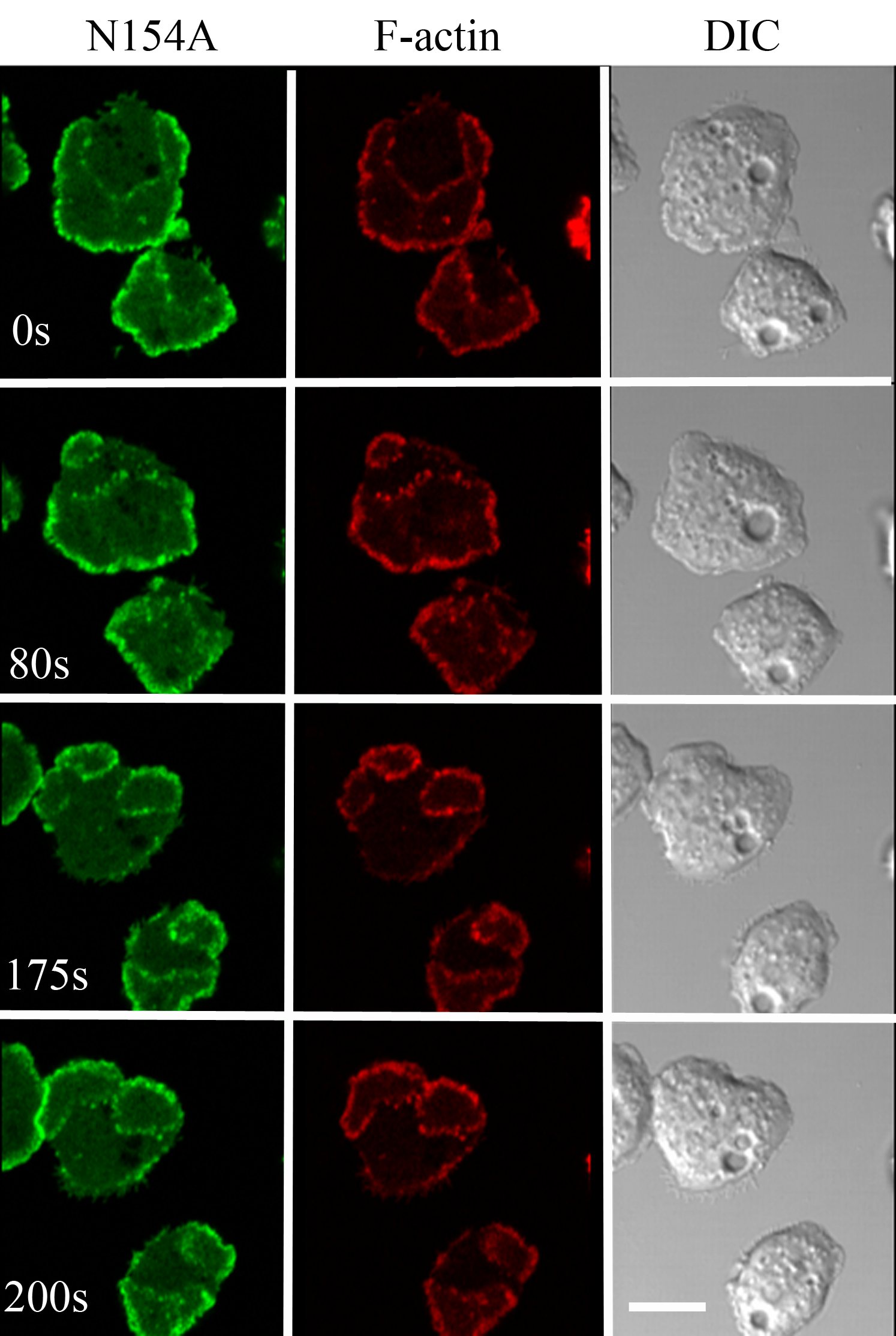

Supplement: Figure S2 — Co-localization of N154A and F-actin waves in live cells; example of different shapes of waves formed in the same cells. myoB−-cells expressing mRFP-lifeact and GFP-N154A (see Fig. 6) were starved for 30 min after which 1 µM latrunculin was added and cell images were recorded at the indicated times (seconds). 0 s corresponds to the beginning of recording. At all stages N154A co-localized with actin waves. This figure corresponds to Movie S1. Bar is 10 µm. (TIF) [file pone.0094306.s002.tif]

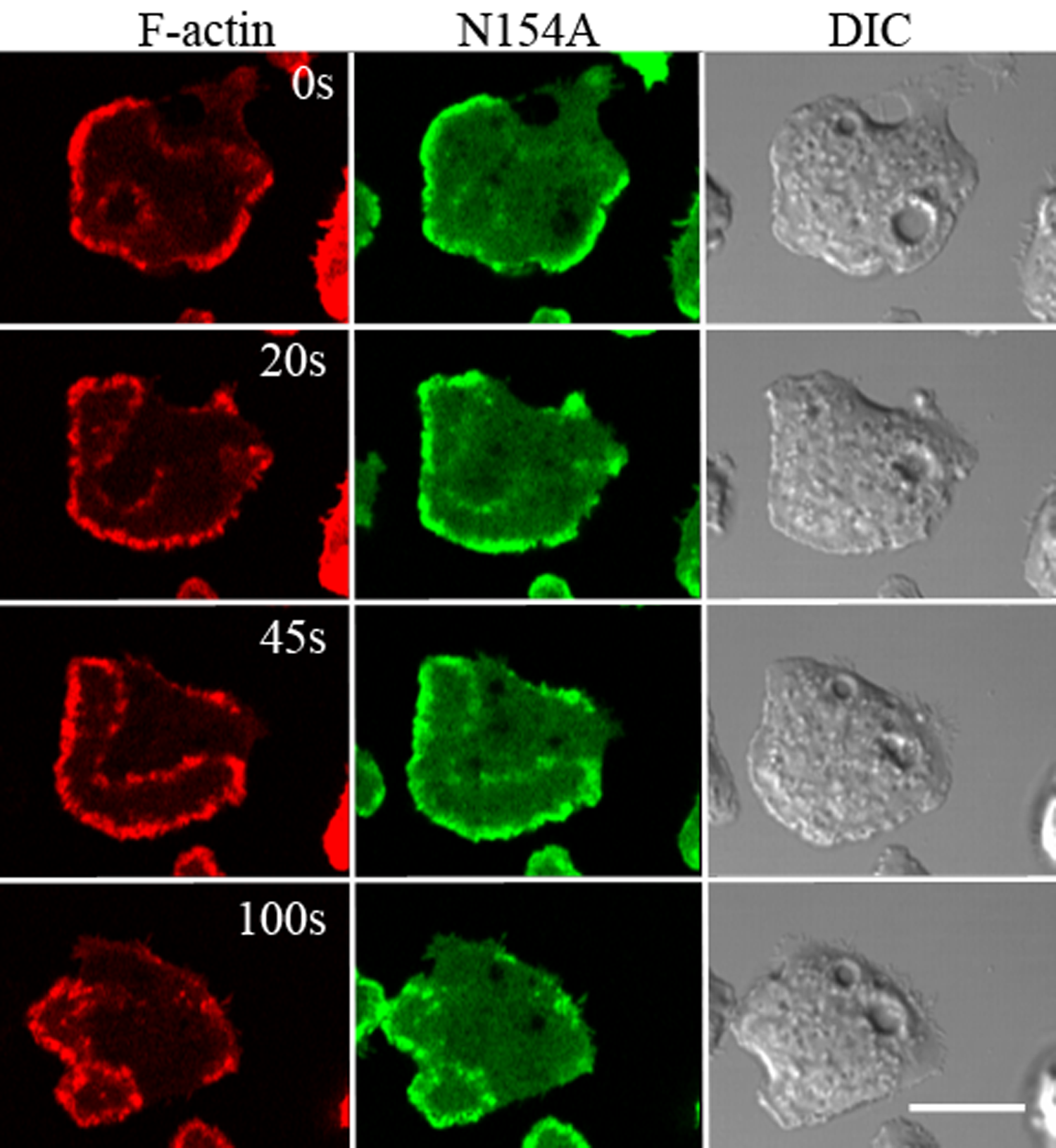

Supplement: Figure S3 — Co-localization of N154A and F-actin in actin waves in live cells; formation of a circular wave in the middle of a cell. myoB−-cells expressing mRFP-lifeact and GFP-N154A (see Fig. 6) were starved for 30 min after which 1 µM latrunculin was added and cell images were recorded at the indicated times (seconds). 0 s corresponds to the beginning of recording. A circular wave formed in the middle of the cell, merged with a peripheral wave and eventually formed a small circular wave at the cell periphery. N154A co-localized with the F-actin wave at all stages. Fluorescence of N154A was weaker at the early stages of wave formation and gained strength with wave expansion. This figure corresponds to Movie S2. Bar is 10 µm. (TIF) [file pone.0094306.s003.tif]

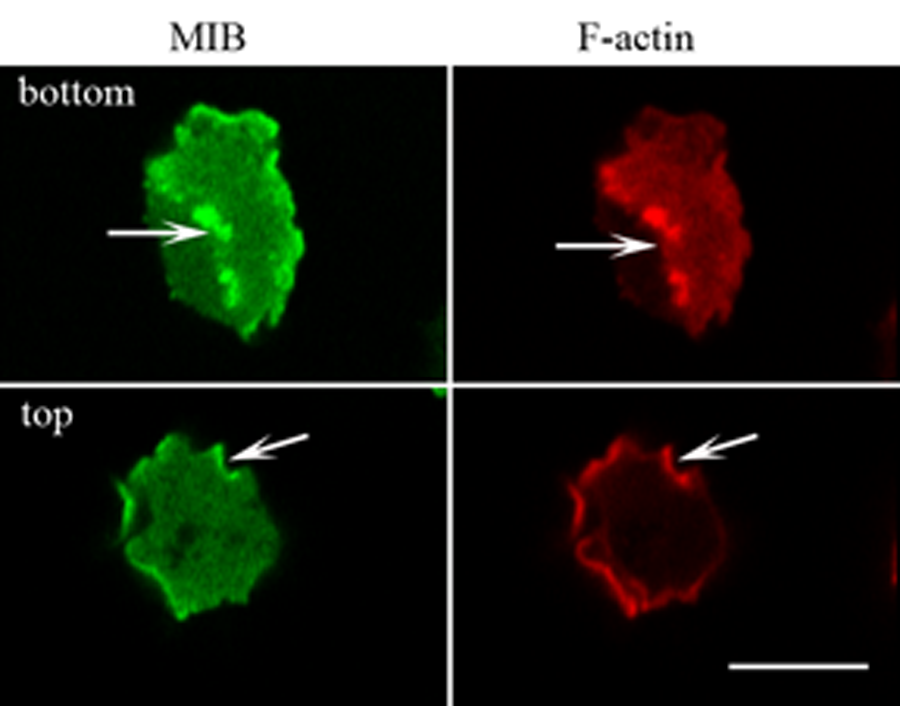

Supplement: Figure S4 — Actin waves and cell protrusions are separated in the Z dimension. myoB−cells co-transfected with GFP-MIB and mRFP-lifeact were starved for 2 h and images were taken at the bottom and top (separated by 7 µm) of a live cell. Arrows point to the wave at the cell bottom and to a protrusion at the top of the same cell. Bar is 10 µm. (TIF) [file pone.0094306.s004.tif]
